# Pyrene–nucleobase conjugates: synthesis, oligonucleotide binding and confocal bioimaging studies

**DOI:** 10.3762/bjoc.13.249

**Published:** 2017-11-28

**Authors:** Artur Jabłoński, Yannic Fritz, Hans-Achim Wagenknecht, Rafał Czerwieniec, Tytus Bernaś, Damian Trzybiński, Krzysztof Woźniak, Konrad Kowalski

**Affiliations:** 1 Faculty of Chemistry, Department of Organic Chemistry, University of Łódź, Tamka 12, PL-91403 Łódź, Poland; 2 Institute of Organic Chemistry, Karlsruhe Institute of Technology, Fritz-Haber-Weg 6, 76131 Karlsruhe, Germany; 3 Universität Regensburg, Institut für Physikalische und Theoretische Chemie, Universitätsstraße 31, D-93040 Regensburg, Germany; 4 Nencki Institute of Experimental Biology, Polish Academy of Sciences, ul. Pasteura 3, 02-093 Warsaw, Poland; 5 Faculty of Chemistry, Biological and Chemical Research Centre, University of Warsaw, Żwirki and Wigury 101, 02-089 Warszawa, Poland

**Keywords:** confocal microscopy, luminescence, nucleobases, oligonucleotide binding, pyrene, X-ray

## Abstract

Fluorescent pyrene–linker–nucleobase (nucleobase = thymine, adenine) conjugates with carbonyl and hydroxy functionalities in the linker were synthesized and characterized. X-ray single-crystal structure analysis performed for the pyrene–C(O)CH_2_CH_2_–thymine (**2**) conjugate reveals dimers of molecules **2** stabilized by hydrogen bonds between the thymine moieties. The photochemical characterization showed structure-dependent fluorescence properties of the investigated compounds. The conjugates bearing a carbonyl function represent weak emitters as compared to compounds with a hydroxy function in the linker. The self-assembly properties of pyrene nucleobases were investigated in respect to their binding to single and double strand oligonucleotides in water and in buffer solution. In respect to the complementary oligothymidine T_10_ template in water, compounds **3** and **5** both show a self-assembling behavior according to canonical base–base pairing. However, in buffer solution, derivative **5** was much more effective than **3** in binding to the T_10_ template. Furthermore the adenine derivative **5** binds to the double-stranded (dA)_10_–T_10_ template with a self-assembly ratio of 112%. Such a high value of a self-assembly ratio can be rationalized by a triple-helix-like binding, intercalation, or a mixture of both. Remarkably, compound **5** also shows dual staining pattern in living HeLa cells. Confocal microscopy confirmed that **5** predominantly stains mitochondria but it also accumulates in the nucleoli of the cells.

## Introduction

**Figure 1:**
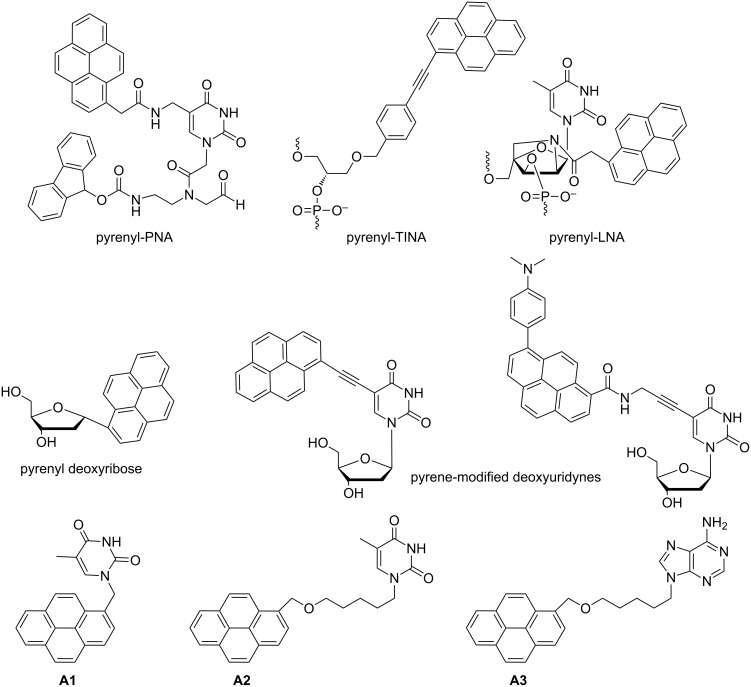
Examples of pyrene derivatives with relevance to nucleic acid chemistry and structures of pyrenyl–nucleobase conjugates **A1**–**A3**.

Pyrene is a planar, polycyclic aromatic hydrocarbon which shows well characterized environment-dependent fluorescence. This property, together with the facile synthetic accessibility, makes it and its derivatives useful for a number of applications, e.g., as materials for organic electronics [1], dyes for mechanochromic materials [2], and fluorescent monomers for polymer synthesis [3]. Pyrenyl derivatives have also attracted considerable attention as fluorescent probes in nucleic acid chemistry and closely related research areas. In particular the pyrene scaffold has been utilized for the construction of abiotic oligopyrenotides with nucleic acid-like structural properties [4], pyrene-modified peptide nucleic acids (PNA) [5], locked nucleic acids (LNA) [6–7], invader LNA [8], and twisted intercalating nucleic acids (TINA) [9]. Furthermore pyrene-modified nucleotides have been used for the construction of DNA-based multichromophore systems [10–13], as cancer detecting markers [14], as fluorescent DNA probes [15], non-covalent binders to canonical oligonucleotide templates [16], and antiviral agents [17–18]. Notably, pyrene excimer formation in DNA template assemblies is much less efficient than in normal pyrene conjugates due to the helical twist between chromophores [19–21]. This helical twist was evidenced by circular dichroism, in particular a strong bisignate Cotton effect for the DNA-templated pyrene assemblies [19–20]. Figure 1 shows selected examples of pyrene-modified nucleic acids and nucleosides.

On the other hand, pyrene–nucleobase conjugates of the general structure pyrene–spacer–nucleobase (Figure 1) have been investigated to a lesser extent than their oligomeric counterparts. A literature survey shows that pyrene–thymines **A1** and **A2** (Figure 1) were utilized as selective fluorescent chemosensors for Hg(II) ions [19,22]. The molecular mechanism of sensing involves Hg(II) ion coordination to two thymine moieties followed by pyrene excimer formation [19]. Furthermore, compound **A2** and adenine derivative **A3** were reported to act as fluorescent sensors for thymine and adenine [23]. To the best of our knowledge, pyrene–nucleobases have not been investigated towards application as fluorescent cell imaging bioprobes so far. Furthermore, self-assembly studies of pyrene–nucleobases on oligonucleotide templates have not been reported as well. The work presented herein addresses these two problems. Accordingly, in this contribution we report on the synthesis, DFT calculations, photophysical characterization, oligonucleotide binding studies, and confocal microscopy studies of the novel pyrene–nucleobase conjugates **2**–**5** (nucleobase = thymine (**2** and **4**), and adenine (**3** and **5**)). Our compounds represent a simple bifunctional design combining the fluorescent reporting pyrenyl group and hydrogen-bonding biological nucleobase vector to be tested in DNA recognition and bioimaging applications.

## Results and Discussion

**Scheme 1:**
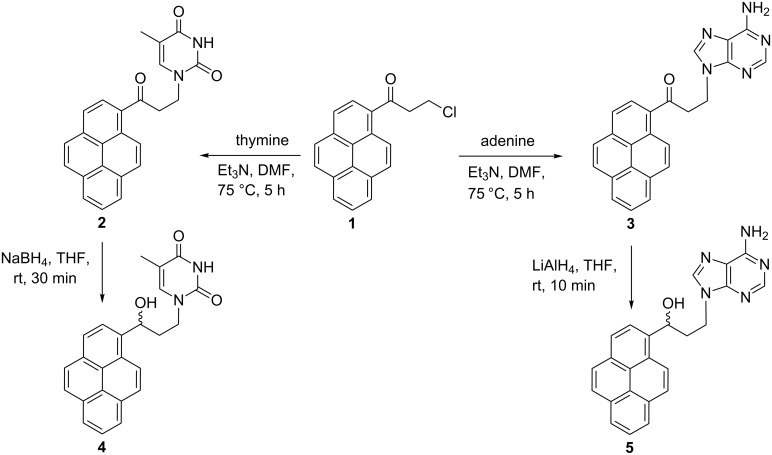
Synthesis of pyrene–nucleobase conjugates **2**–**5**.

A straightforward, one-pot two-step methodology for aryl–nucleobases has been recently developed in our laboratory and was examined in respect to various aryl starting materials [24–26]. In this work, the starting material 1-(3-chloropropionyl)pyrene (**1**), was obtained via Friedel–Crafts reaction of pyrene with 3-chloropropionyl chloride [27]. Subsequently, the pyrenyl–nucleobase conjugates **2** and **3** were obtained by reactions of 1-(3-chloropropionyl)pyrene (**1**) with thymine and adenine, respectively (Scheme 1).

After work-up, compounds **2** and **3** were isolated as yellow solids in 54% and 56% yields, respectively. In the following step, the carbonyl function in **2** was reduced with sodium borohydride to afford alcohol **4** as a colorless solid in 91% yield. Surprisingly, an attempt to reduce the carbonyl function in adenine derivative **3** failed. The reaction performed at the same conditions as for **2** afforded a complex mixture of products. In order to solve this problem, lithium aluminum hydride was used as reducing agent. In this case, the reaction proceeded smoothly to yield **5** as colorless solid in 85% yield (Scheme 1). All pyrenyl derivatives **1**–**5** were characterized by ^1^H NMR, ^13^C NMR, and IR spectroscopy, mass spectrometry and elemental analysis, and the analytical data confirmed the proposed structures. Furthermore the molecular structure of pyrenyl–thymine **2** was confirmed by single-crystal X-ray analysis.

### Crystal structure

**Figure 2:**
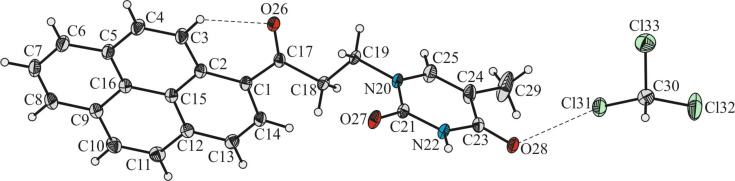
ORTEP diagram of **2** at 50% probability level. The hydrogen and halogen bonds are represented by dashed lines. Selected bond lengths (Å) and angles (°): O26–C17, 1.223(2); O27–C21, 1.233(2); O28–C23, 1.225(2); N20–C25, 1.380(2); N22–C21, 1.372(2); N22–C23, 1.389(2); C1–C17, 1.501(2); C1–C2, 1.428(2); C15–C16, 1.433(2); C12–C13, 1.399(2); C30–Cl31···O28, 2.972(2); C3–H3···O26, 2.879(2); C1–C17–O16, 123.78(15); N22–C23–C24, 114.79(15); O27–C21–N22, 121.79(15); O27–C21–N20, 122.60(15); N20–C21–N22, 115.61(14); C2–C1–C17, 123.28(15).

Crystals of **2** suitable for X-ray diffraction analysis were obtained by slow diffusion of pentane into a chloroform solution of **2**. The oak ridge thermal-ellipsoid plot (ORTEP) with the atom labelling scheme is shown in Figure 2, together with selected bond lengths and angles. Crystal and structure refinement data are given in Supporting Information File 1 (Table S1).

**Figure 3:**
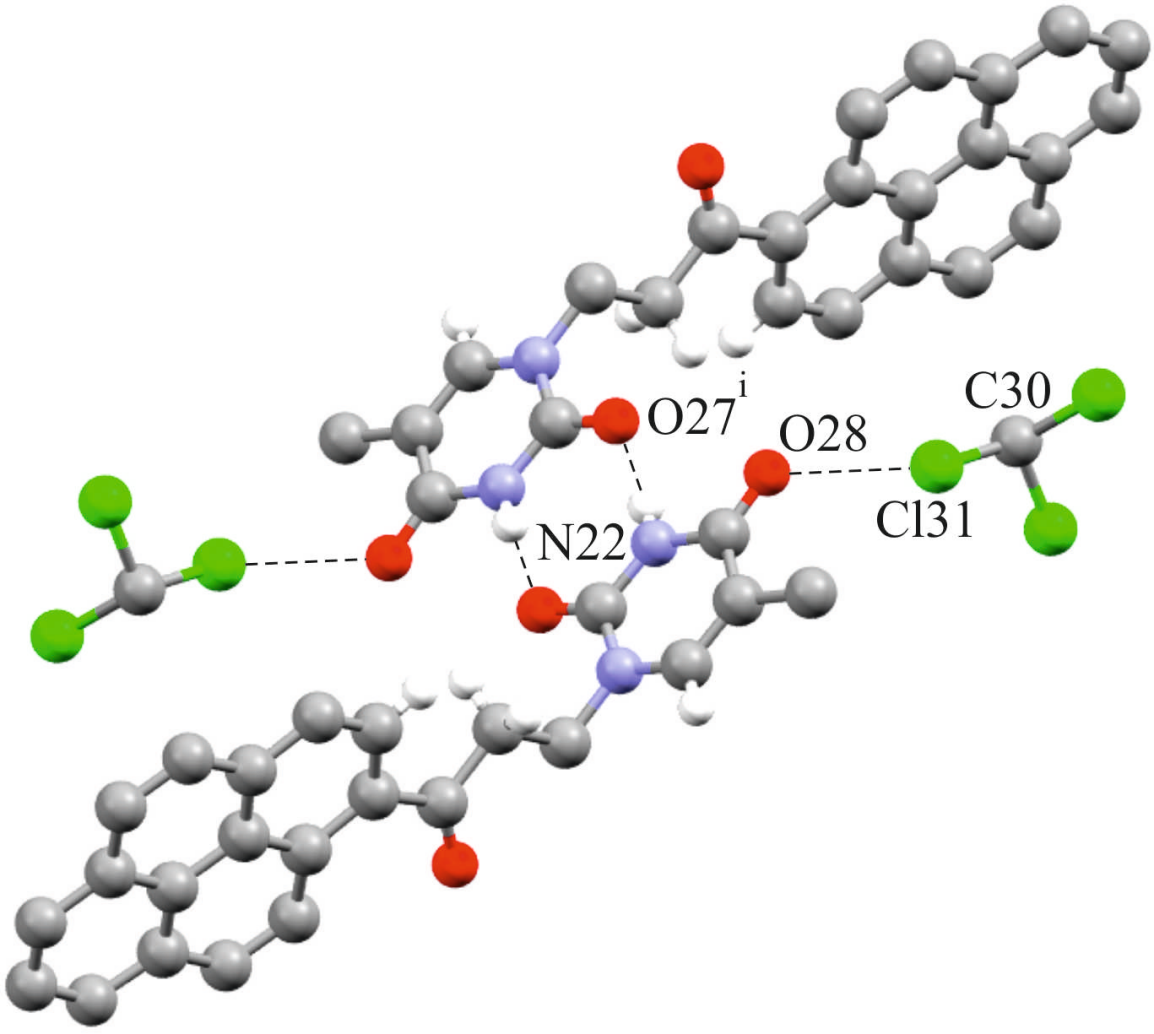
Intermolecular hydrogen bonding (N22–H22···O27 distance = 2.882(2) Å) and halogen bonding (C30–Cl31···O28 distance = 2.972(2) Å) observed in the crystal packing of **2**.

Compound **2** crystallizes as a chloroform solvate in the monoclinic *I*2/*a* space group with one pair of given molecules in the asymmetric part of the unit cell. The crystallographic structure confirms that the C1-substituted pyrene is connected to the N20 atom of the thymine moiety through the 1-oxopropionyl linker. The geometry of the pyrenyl moiety deviates from planarity of 0.039 Å [28] while the average deviation from planarity for the thymine group is 0.011 Å. The planes delineated by the non-hydrogen atoms of substituted pyrene and thymine ring systems, are oriented at 84.01(2)° to each other. In the crystal the carbonyl functionality bond C17=O26 is tilted from the plane of the pyrenyl moiety. Accordingly, the mean planes delineated by the C1/C17/C18/O26 atoms and non-hydrogen atoms of the pyrenyl group are inclined by an angle of 17.26(5)°. The carbonyl oxygen atom O26 is involved in the intramolecular hydrogen bond with the hydrogen atom H3 of the pyrenyl group. The C3–H3···O26 hydrogen bond length is 2.879(2) Å. In the solid state, each independent molecule of compound **2** forms a dimeric structure stabilized through hydrogen bonds between the N22–H22 amido and the C21=O27 carbonyl function of the thymine moieties (Figure 3). The N22···O27 distance is 2.882(2) Å while the N22–H22···O27 angle is 174(3)°. Similar dimeric systems have been observed in the molecular structures of the metallocene–nucleobase derivatives [24,29]. In addition, each molecule of **2** in the dimer is further involved in a Cl···O halogen bond with an adjacent molecule of chloroform (Figure 3).

Further details of intermolecular interactions present in the crystal structure of **2** and full list of bond lengths and angles are given in Supporting Information File 1 (Table S3, Table S4, and Figure S6).

### Photophysical characterization and DFT calculations

**Figure 4:**
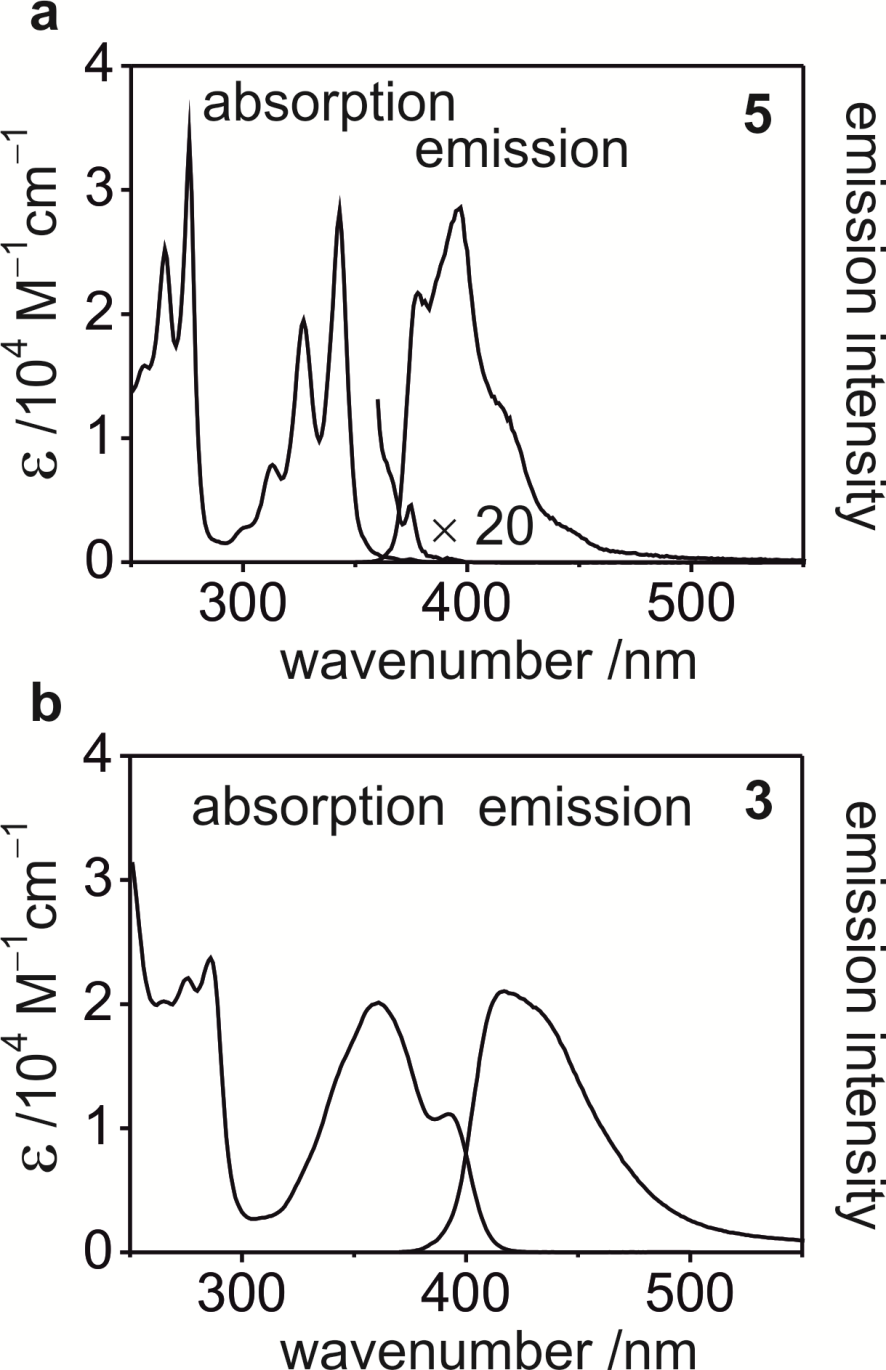
UV–vis absorption and fluorescence spectra of pyrene–adenines **5** (a) and **3** (b) in diluted (*c* ≈ 10^−5^ M) dichloromethane solutions at ambient temperature.

UV–vis absorption and fluorescence properties of pyrene–nucleobase conjugates **2**–**5** were characterized in dichloromethane at ambient temperature. The absorption and emission spectra of the adenine derivatives **3** and **5** are reproduced in Figure 4.

The photophysical properties of **5** (Figure 4a) resemble the properties of unsubstituted pyrene [30]. In the low energy UV and blue spectral region between 250 and 400 nm, three well-resolved structured absorption bands are seen. The lowest energy band due to the transition to the lowest excited singlet state S_1_ at 375 nm has a small molar absorption coefficient of ε = 230 M^−1^ cm^−1^ (Table 1).

**Table 1:** UV–vis absorption and emission data for pyrene–adenine conjugates **2**–**5** measured in diluted dichloromethane solution at ambient temperature.

compound	absorption maximum /nm (molar absorption coefficient /M^−1^ cm^−1^)			fluorescence maximum /nm	decay time τ_f_ /ns	quantum yield 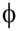 _f_

	S_3_ ← S_0_	S_2_ ← S_0_	S_1_ ← S_0_			
				
**2**	286 (1.7 × 10^4^)	367 (1.1 × 10^4^)	396 (0.8 × 10^4^)	425	<2	<2%
**3**	286 (2.4 × 10^4^)	360 (2.0 × 10^4^)	393 (1.1 × 10^4^)	417	<2	<2%
**4**	277 (3.8 × 10^4^)	344 (3.3 × 10^4^)	375 (2.9 × 10^2^)	377, 397^a^	150	37%
**5**	276 (3.8 × 10^4^)	343 (3.4 × 10^4^)	375 (2.3 × 10^2^)	378, 397^a^	160	40%

^a^Maxima of the partly resolved vibronic progressions. Emission spectrum for **5** is reproduced in Figure 3.

This is due to the symmetry forbidden S_1_ ↔ S_0_ electronic transition in pyrene. At 343 nm (≈29160 cm^−1^) and 276 nm (≈36230 cm^−1^) absorption maxima corresponding to electronic transitions to higher energy excited singlet states S_2_ ← S_0_ and S_3_ ← S_0_, respectively, are seen. These maxima are accompanied by further maxima of respective vibrational bands, for instance at 327 nm (≈30580 cm^−1^) and 312 nm (≈32050 cm^−1^) with the vibronic progression energy of Δ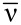 ≈ 1450 cm^–1^, for the S_2_ ← S_0_ transition. The S_2_ ← S_0_ and S_3_ ← S_0_ transitions, with molar absorption coefficients of ε = 2.8 × 10^4^ M^−1^cm^−1^ (at 343 nm) and 3.4 × 10^4^ M^−1^cm^−1^ (at 276 nm), respectively, are strongly allowed and correspond to the S_2_ ← S_0_ and S_3_ ← S_0_ transitions of unsubstituted pyrene.

Pyrene–adenine **5** shows strong deep blue fluorescence in diluted dichloromethane solution. The emission spectrum remains partly resolved with apparent vibronic maxima at 378 and 397 nm (Figure 4a, Table 1). Thus, the blue flank of the emission band overlaps with the S_1_ ← S_0_ absorption band at 375 nm which is the *E*_00_, the energy that is gained by excitation. In degassed solution, the emission decays with a decay constant of τ_f_ = 160 ns at a quantum yield of 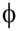
_f_ = 40%. These values correspond to a fluorescence rate k^f^ = 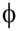
_f_/τ_f_ = 2.5 × 10^6^ s^−1^. This relatively slow radiative decay rate k^f^ again shows that the S_1_ ↔ S_0_ electronic transitions are forbidden, in accordance with the small molar absorption coefficient (230 M^−1^ cm^−1^) found for the S_1_ ← S_0_ transition in the absorption spectrum.

Pyrene–thymine **4** shows similar absorption and emission behavior to the pyrene–adenine **5** that closely resemble the properties of unsubstituted pyrene (Table 1). These results show that the two aromatic parts – pyrene and nucleobase – of conjugates **4** and **5** are not electronically coupled.

The absorption and emission properties of pyrene–carbonyl derivatives **2** and **3** significantly differ from that described above for hydroxy derivatives **4** and **5** (Figure 4b, Table 1). In particular, the lowest absorption bands of **2** and **3** with measured maxima at 396 and 393 nm, respectively, show symmetry allowed character (ε = 1.1 × 10^4^ M^−1^cm^−1^). They overlap with the next bands (unresolved) centered at 367 and 360 nm, respectively. The observed changes relative to compounds **4** and **5** and unsubstituted pyrene reflect distinct electronic structure changes of the chromophoric fragment induced by extension of the aromatic system due to conjugation with the carbonyl groups.

The pyrene carbonyls **2** and **3** show distinctly weaker fluorescence than the pyrene alcohols **4** and **5**. The quantum yields at ambient temperature are at least 20 times lower than for **4** and **5**. The decay times drop to below 2 ns (Table 1). Similar trends were already observed for 1-acetylpyrene [31]. In the latter case, low fluorescence intensity and fast decay of fluorescence were rationalized by the presence of low-energy nπ* excited states. In particular, for the lowest excited singlet state ^1^ππ* efficient intersystem crossing to a triplet state ^3^nπ* close in energy can be expected according to the El-Sayed rule. Then, this ^3^nπ* state decays nonradiatively to the ground state, directly or via internal conversion to lower triplet states. Thus, the ^3^nπ* state provides a path for efficient depopulation of the emissive ^1^ππ* singlet state and, thus, for quenching of fluorescence. In the hydroxy derivatives **4** and **5**, such low-energy nπ* states are not present. This explanation is further substantiated using time-dependent density functional (TD-DFT) computations for pyrene–adenine conjugates **3** and **5**.

The molecular structures of compounds **3** and **5** were optimized at the B3LYP/6-311G(d,p) theory level. For the ground state geometry, ten excitations, five without spin flip and five to triplet excited states, respectively, were computed. The results for luminescence relevant transitions are summarized in Table 2.

**Table 2:** Selected lowest-energy vertical electronic transitions resulting from TD-DFT calculations for pyrene–adenine conjugates **3** and **5** in the ground state geometry at the B3LYP/6-311G(d,p) theory level. “Holes” (starting orbitals) and “electrons” (final orbitals) represent natural transition orbitals [32–33] describing each excited state.

compound **3**					

electronic transition	transition energy	oscillator strength	natural transition orbitals		character

			hole	electron	
				
S_0_ → T_1_	2.03 eV	0	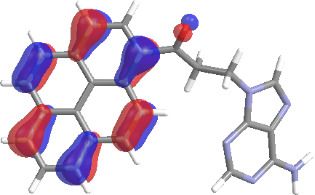	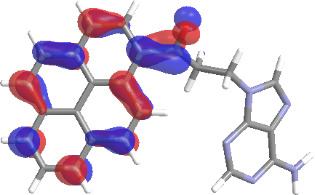	^3^ππ*
S_0_ → T_2_	3.17 eV	0	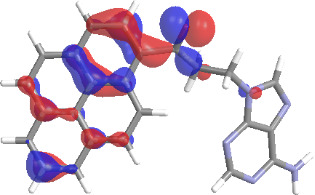	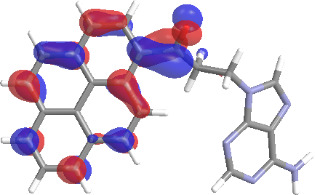	^3^nπ/ππ*
S_0_ → S_1_	3.35 eV	0.302	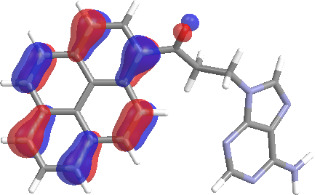	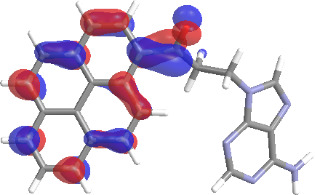	^1^ππ*

compound **5**					

electronic transition	transition energy	oscillator strength	natural transition orbitals		character

			hole	electron	
				
S_0_ → T_1_	2.21 eV	0	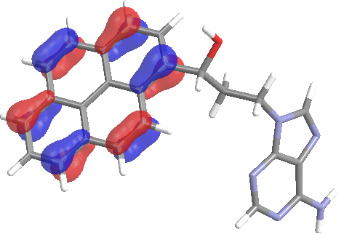	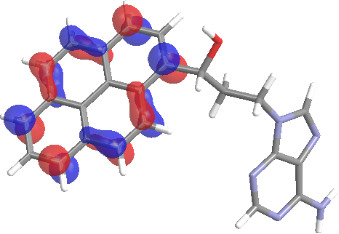	^3^ππ*
S_0_ → T_2_	3.41 eV	0	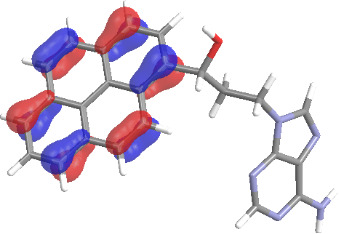	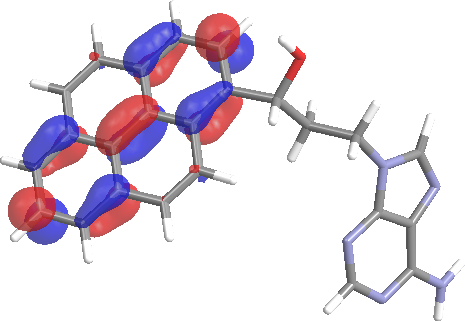	^3^ππ*
S_0_ → T_3_	3.52 eV	0	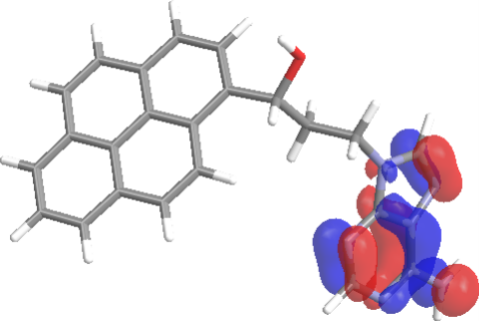	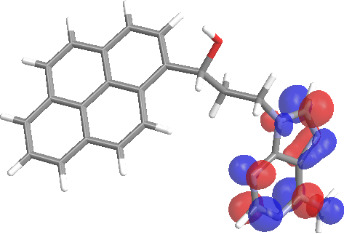	^3^ππ*
S_0_ → T_4_	3.54 eV	0	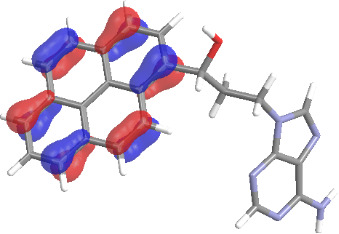	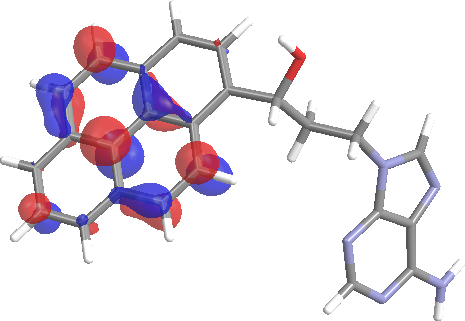	^3^ππ*
S_0_ → T_5_	3.56 eV	0	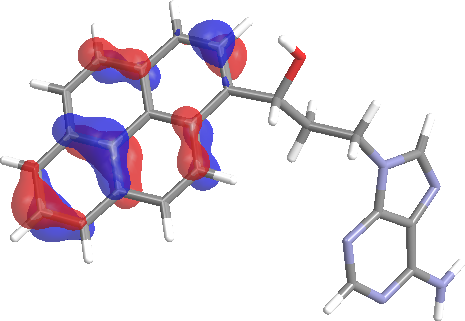	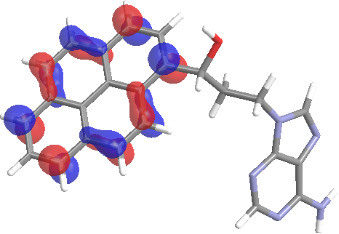	^3^ππ*
S_0_ → S_1_	3.59 eV	0.305	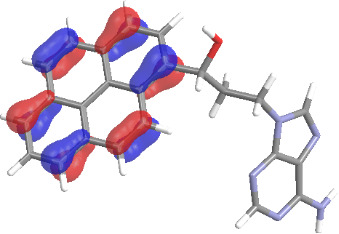	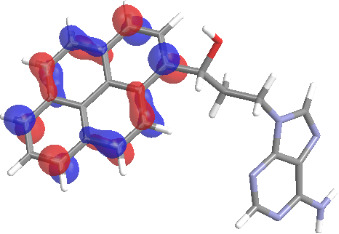	^1^ππ*

The TD-DFT computations predict, in particular, that the fluorescent state S_1_ of hydroxy compound **5** with the calculated transition energy for S_0_ → S_1_ excitation of 3.59 eV lies 0.24 eV higher in energy than the S_1_ state of the carbonyl **3**. This S_1_ energy difference resembles the spectral differences observed for the two compounds, i.e., the substantial red shift of the lowest absorption band and fluorescence of the carbonyl compound **3** as compared to the hydroxy compound **5**. The computations reveal several triplet states below the lowest singlet excited state. In particular, the carbonyl pyrene derivative **3** displays a triplet state of nπ* character being lower in energy than the emissive singlet state S_1_ (being a pyrene ^1^ππ* state). Thus, intersystem crossing between these two states can be efficient giving rise to also efficient radiationless depopulation of the emissive singlet state relative to aliphatic analogues, in that quenching ^3^nπ* states are not present.

### Interactions with oligonucleotides

Compounds **2**–**5** are characterized by structural features originated from their components: a planar pyrenyl group and the heterocyclic nucleobase unit. While the pyrenyl group is known to act as intercalator [8–9], the nucleobases are known to self-assemble via a network of hydrogen bonds [16,34]. Our aim was to investigate the interactions between the compounds **2**–**5** when they bind specifically to a given DNA template. The titration experiments were performed with single and double-stranded oligonucleotides.

**Figure 5:**
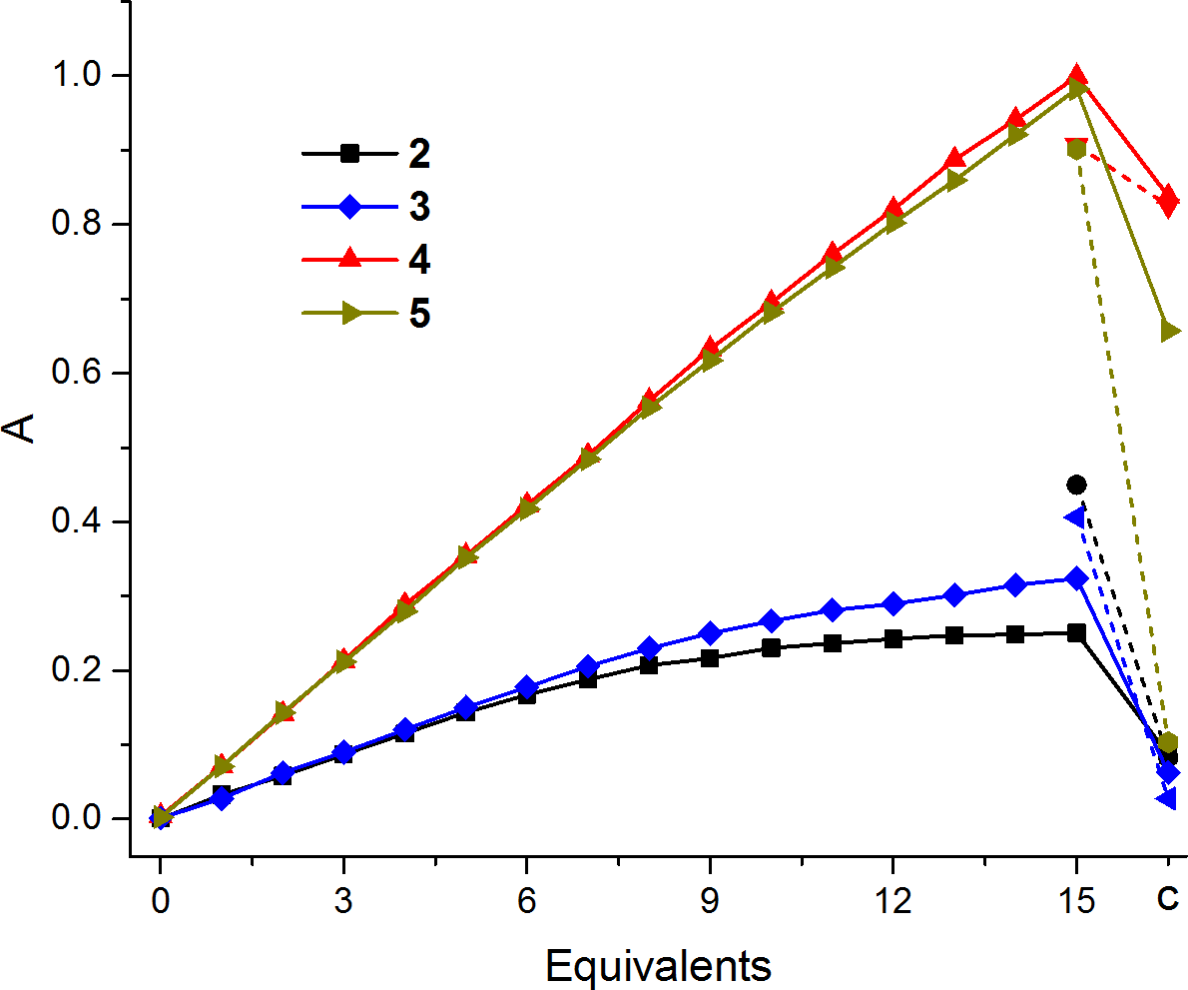
Absorption changes during titration of **2** and **4** (λ = 344 nm) in the presence of (dA)_10_, and **3** and **5** (λ = 364 nm) in the presence of T_10_ (1.25 μM template, 15 equiv = 18.8 μM) in H_2_O. After complete titration, the unbound chromophores precipitated due to their insolubility in aqueous solution, the pellet was removed and the supernatant samples showed a weaker pyrene absorption (c) that corresponds to the amount of the template assembled pyrenes that were kept in solution. Dashed lines show control experiments without the templates (dA)_10_ or T_10_, respectively.

The interactions between the chromophores and a 10-mer of the single-stranded oligo-2’-deoxyadenosine, (dA)_10_, or oligothymidine, T_10_, respectively, as template strands were investigated in water. Due to the nearly complete insolubility of the chromophore–nucleobase conjugates in water it is possible to follow this self-assembly simply by UV–vis absorption spectroscopy (Figure 5).

Only those chromophore–nucleoside conjugates that are bound and assembled along the (dA)_10_ or T_10_ template are kept soluble in aqueous media. A higher concentrated stock solution of each chromophore–nucleobase conjugate was prepared in DMSO and added as aliquots to an aqueous solution (2.5 μM) of the (dA)_10_ or T_10_ template. The volume of the aliquots is small (μL range) that the DMSO concentration in the final samples never exceeded 5%. According to our previous studies, a low concentration of DMSO is tolerated by the helical DNA conformation [16]. After centrifugation, the unbound chromophores precipitated due to their insolubility in aqueous solution, the pellet was removed and the supernatant showed a weaker pyrene absorption that corresponds to the amount of the template assembled pyrenes that were kept in solution. These experiments revealed an average self-assembly grade of 29% for **3** and 74% for **5** with respect to the 10 available binding sites on the template strand T_10_. Conjugate **2** showed no self-assembly at all to the (dA)_10_ template and **4** gave no clear results due to its general solubility in water even without the template strand (dA)_10_. These results revealed a clear preference and selectivity of the adenine-conjugated pyrenes **3** and **5** for binding to the template T_10_. The significant higher self-assembly grade of **5** compared to **3** can be assigned to the hydroxy group next to the pyrene that is only present in **5**. The sp^3^-hybridized C-atom next to the pyrenyl moiety results in a higher flexibility for the whole chromophore conjugate and allows more efficient self-assembly, whereas the carbonyl group of **3** induces a more rigid conformation that interferes with self-assembly.

To gain more insight about self-assembly of **3** and **5**, additional titration experiments were done with the double-stranded template (dA)_10_-T_10_ that was prehybridized, so that canonical base pairing of the chromophore–nucleoside conjugates with the template could be excluded. After centrifugation, compound **3** shows no effective self-assembly to single or double-stranded templates in buffer; only in water an assembly occurring to both single stranded templates (Table 3).

**Table 3:** Self-assembly ratios of **3** and **5** with single-stranded templates (dA)_10_, T_10_ and double strand (dA)_10_-T_10_ under pure aqueous (no salts) and buffer conditions (50 mM NaP_i_, 250 mM NaCl, pH 7). The self-assembly ratio was determined by UV–vis absorption after centrifugation as described in the text and describes the number of assembled pyrene moieties with respect to the number of binding sites at the template, e.g., 10 for T_10_).

template	solvent	assembly grade with **3** (%)	assembly grade with **5** (%)

(dA)_10_-T_10_	buffer	0.4	112
(dA)_10_	buffer	1.3	65
(dA)_10_	H_2_O	35	0
T_10_	buffer	3.3	86
T_10_	H_2_O	29	74

Compound **5** shows a significantly higher self-assembly ratio to T_10_ than to (dA)_10_, indicating canonical base pairing. Furthermore, the self-assembly ratio (number of assembled pyrene moieties with respect to the number of binding sites at the template, e.g., 10 for T_10_) of 112% to the double-stranded template is significantly higher compared to the single-stranded templates and must be assigned either to a triple-helix-like binding or intercalation, or a mixture of both (Table 3).

In summary, compounds **3** and **5** show a self-assembling behavior in the presence of the complementary DNA template strand according to canonical base pairing rules. In addition, the self-assembly grades of **5** indicate both a canonical base pairing and another, likely intercalative, self-assembly binding mechanism. This conclusion is supported by the fact that **5** more selectively binds to T_10_ compared to (dA)_10_ and that it shows a self-assembly grade of over 100% to a double-stranded template, which excludes canonical base pairing. An interesting side effect is also that the self-assembly process is essentially influenced by the buffer salts. Especially the more flexible derivate **5** assembles better to single-stranded T_10_ and double-stranded (dA)_10_-T_10_.

### Confocal microscopy

**Figure 6:**
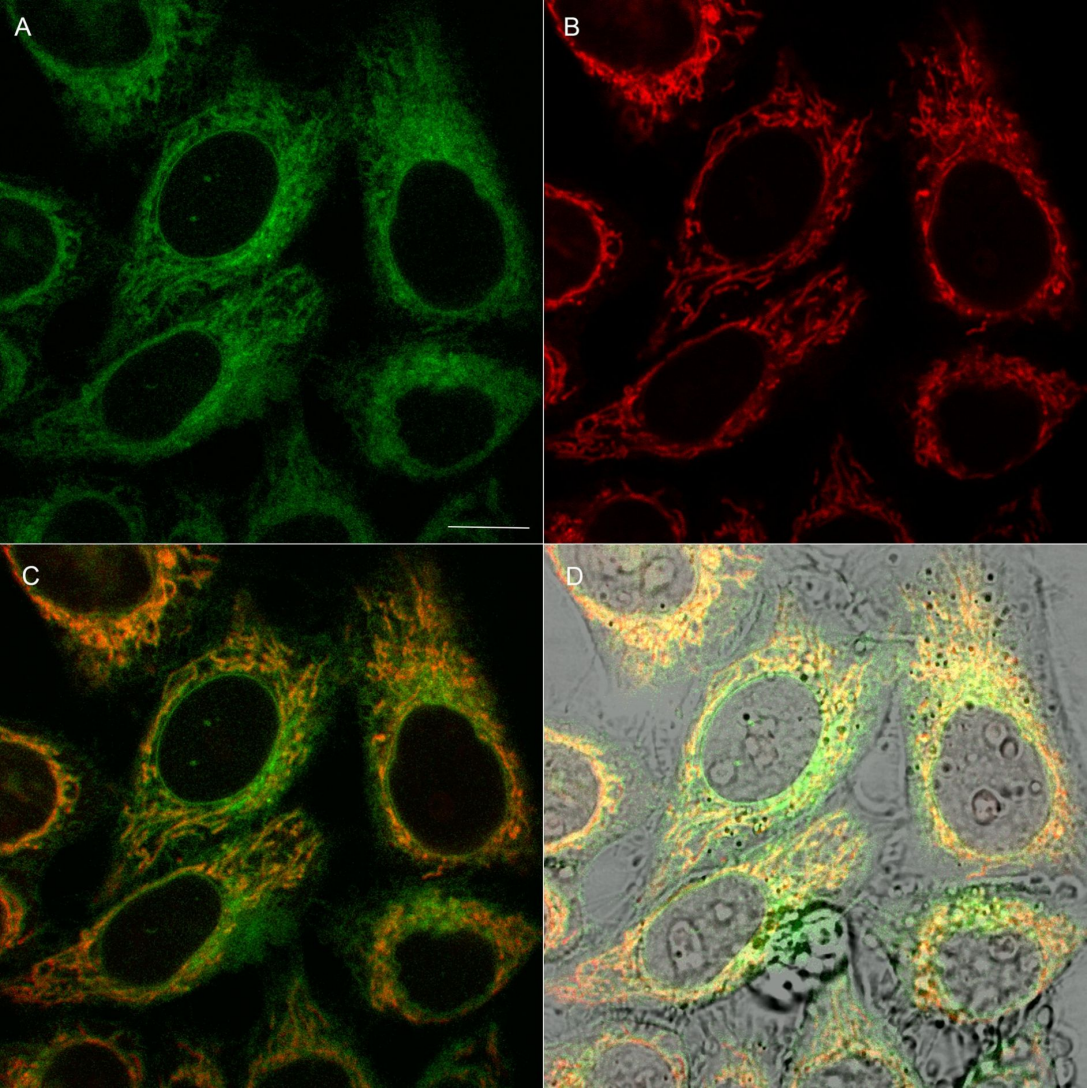
Cellular distribution of **4** in living HeLa cells. (A) Fluorescence of **4** (green). (B) Fluorescence of mitochondria-specific MitoTracker Red^®^ (red). (C) Merged image of **4** (green) and MitoTracker (red). (D) Merged image of (C) and transmitted light (gray). Scale bar – 10 µm.

**Figure 7:**
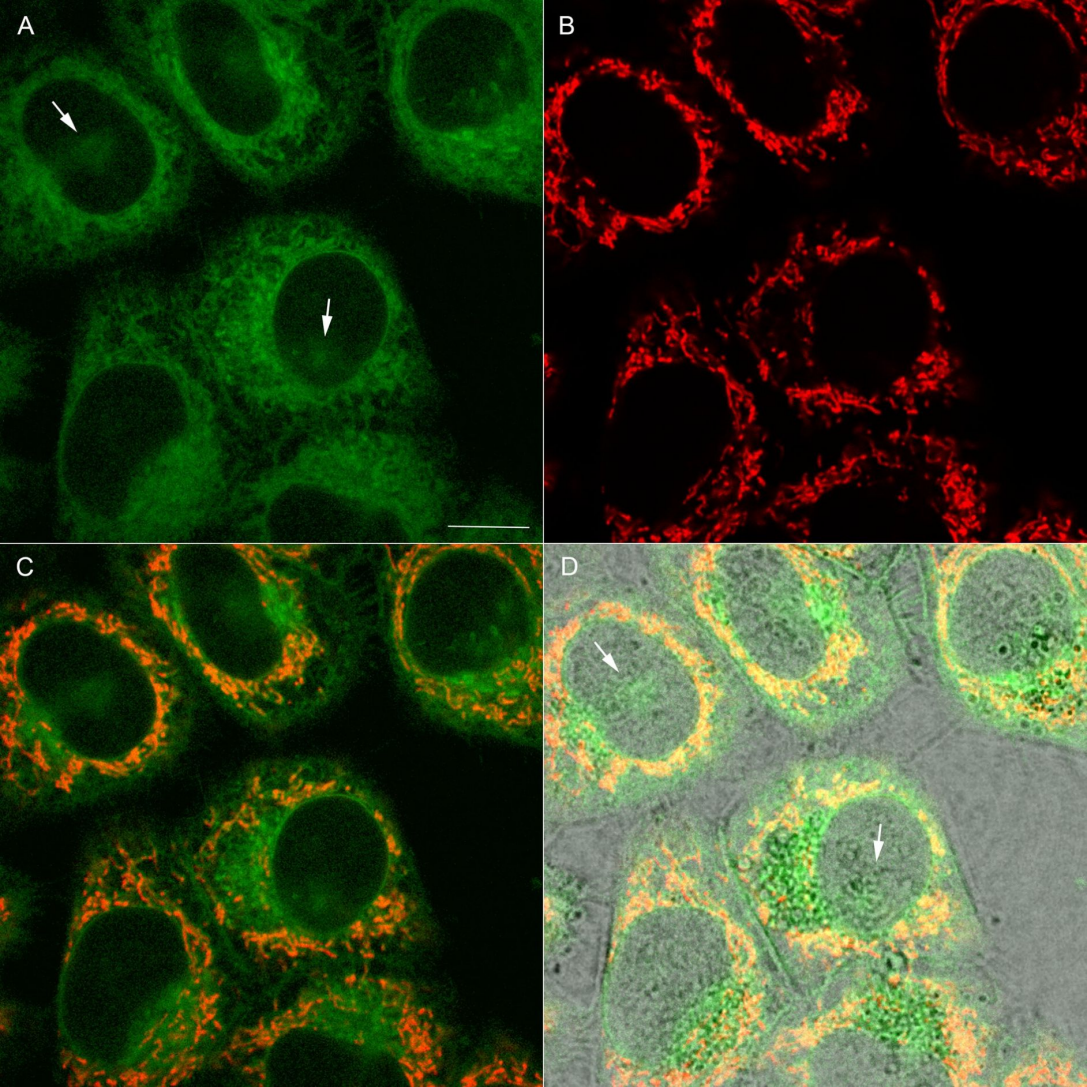
Cellular distribution of **5** in living HeLa cells. (A) Fluorescence of **5** (green). Arrows are marking the regions corresponding to nuclear staining. (B) Fluorescence of mitochondria-specific MitoTracker Red^®^ (red). (C) Merged image of **5** (green) and MitoTracker (red). (D). Merged image of (C) and transmitted light (gray). Scale bar – 10 µm.

Over the past years, there has been an increased interest in the development of luminescent probes for bioimaging. In that respect, both metal-containing [35] as well as organic [36] luminophores were examined. We found that, due to significant phototoxic activity, compounds **2** and **3** were not compatible with live cell imaging. On the other hand, accumulation of **4** and **5** was detectable with confocal microscopy, following 15 min incubation of live human cancer HeLa cells with 200 nM of each compound. Both **4** and **5** produced granular staining pattern (Figure 6A and Figure 7A, respectively), which indicate their mitochondrial localization.

Furthermore, a weaker (diffuse) emission of **4** and **5** was observed in cytoplasm and plasma membranes of HeLa cells. The mitochondrial staining was confirmed by a co-localization experiment with MitoTracker Red^®^, an established fluorescent mitochondrial marker (Figure 6BC and Figure 7BC, respectively). To quantify the co-localization of **4** and **5** dyes and MitoTracker Red^®^ the Manders (MCC) and Pearson (PCC) correlation coefficient were used [37]. The calculated values of MCC were 0.83 ± 0.12 (for **4**) and 0.71 ± 0.11 (for **5**). Likewise, the average values of PCC were 0.67 ± 0.09 (for **4**) and 0.55 ± 0.06 (for **5**). These data are compatible with preferential, but not exclusive localization of both compounds in mitochondria. This effect is slightly more pronounced (at *p* = 0.05) for **4**, as compared with **5** (Figure 6A and Figure 7A, respectively). Moreover, a minor fraction of HeLa cells (<8%) incubated with **5** exhibited a weak nuclear staining pattern (Figure 7, arrows). The comparison of compound **5** cellular fluorescence with transmitted light image (Figure 7D) indicates that the probe might localize in nucleoli. Noticeably, this pattern was not observed when the cells were labeled with **4**. On the other hand fluorescence emission spectra of both compounds in cells were similar (Figure S7, Supporting Information File 1) and compatible with their counterparts registered in dichloromethane solution. This confirms stability of both luminophores in a complex cellular environment.

## Conclusion

In conclusion, we report on four fluorescent pyrene–nucleobase (nucleobase = thymine, adenine) conjugates. Compounds **2** and **3** were obtained in reactions of 1-(3-chloropropionyl)pyrene with thymine and adenine, respectively. An X-ray crystal structure analysis of derivative **2** shows dimers stabilized by thymine–thymine hydrogen bonds. Conjugates **2** and **3** with the carbonyl function in the linker moiety are characterized by short emission decay times and low quantum yields. The reduction of the carbonyl function in **2** and **3** afforded products **4** and **5** that display long fluorescence decay times (ca. 150 ns) and high emission quantum yields (ca. 40%). The interactions between the adenine derivatives (**3** and **5**) and the single-stranded oligonucleotide templates, (dA)_10_, T_10_ and the double-stranded template (dA)_10_-T_10_ were investigated in water and in buffer solution. The compounds bind to single-stranded templates and the strength of binding is solvent-dependent. In water solution compounds **3** and **5** both self-assemble on T_10_ oligomer according to canonical base–base pairing while in buffered solution only **5** effectively binds to the template. Interestingly, the adenine derivative **5** binds to the double-stranded (dA)_10_-T_10_ template with a self-assembly ratio of 112%. Such a high value of a self-assembly ratio may suggest a differentiated mechanism of self-assembly on the double-stranded template which may involve a triple-helix-like binding, intercalation, or a mixture of both. Confocal microscopy revealed a similar cellular staining pattern for compounds **4** and **5**. Both derivatives predominantly accumulate in mitochondria of living HeLa cells and the adenine conjugate **5** accumulates also in the nucleoli of the cells. Our results substantiate further studies on pyrene–nucleobase conjugates as nucleic acid fluorescent probes and as cell imaging agents.

## Experimental

### General

All preparations were carried out using standard Schlenk techniques. Chromatographic separations were carried out using silica gel 60 (Merck, 230–400 mesh ASTM). Triethylamine, dimethylformamide, and tetrahydrofuran were distilled and deoxygenated prior to use. Other solvents were of reagent grade and were used without prior purification. Thymine, adenine, 3-chloropropionyl chloride, lithium aluminum hydride, sodium borohydride, and aluminium chloride were purchased from commercial suppliers and were used without further purification. ^1^H NMR (600 MHz) and ^13^C{H} NMR (150 MHz) spectra were recorded with a Bruker Avance III 600 spectrometer operating at 298 K in the Fourier transform mode. Chemical shifts are reported in δ units (ppm) using residual DMSO (^1^H δ 2.50 ppm, ^13^C δ 39.70) or CHCl_3_ (^1^H δ 7.26 ppm, ^13^C δ 77.00) as reference. Infrared spectra were recorded with a FTIR Nexus Nicolet apparatus. Mass spectra were recorded with a Varian 500-MS iT mass spectrometer (ESI) or with a Finnigan Mat95 mass spectrometer (EI). Microanalyses were determined by Analytical Services of the Polish Academy of the Sciences, Łódź.

### Experimental details

#### Luminescence measurements

UV–vis absorption spectra were recorded with a Varian Cary 300 double beam spectrometer. Luminescence spectra were measured for air-saturated and degassed diluted (c ≈ 5·10^–5^ M) solutions in ethanol with a Horiba Jobin Yvon Fluorolog 3 steady-state fluorescence spectrometer. For decay time measurements a PicoQuant LDH-P-C-375 pulsed diode laser (λ_exc_ = 372 nm, pulse width 100 ps) was applied as the excitation source. The emission signal was detected with a cooled photomultiplier attached to a FAST ComTec multichannel scalar card with a time resolution of 250 ps. Photoluminescence quantum yields 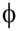
_PL_ were determined with a Hamamatsu C9920-02 system equipped with a Spectralon^®^ integrating sphere.

#### Computational details

Molecular geometries and electronic structures were calculated using the density functional theory (DFT) with the hybrid gradient corrected correlation functional B3LYP [38] and Gaussian-type basis functions 6-311G(d,p) [39]. TD-DFT calculations were performed in the optimized ground state geometry using the same B3LYP functional and basis sets. Five lowest singlet and triplet excitations were computed. All computations were carried out using the Gaussian 09 program package [40].

#### Oligonucleotide self-assembly measurements

The absorption and emission experiments were made at controlled temperature of 20 °C. The absorption was measured with a Lambda 750 from Perkin-Elmer and the fluorescence with a Fluoromax-4 spectrofluorometer from HORIBA-Scientific. For analysis stock solutions of each pyrene derivative in DMSO with a concentration of 1 mM were made. The whole measurements were performed in aqueous solutions without any salts. Blank measurements were made in deionized water. The dyes were added in 1 to 3 equivalent steps compared to the concentration of the template strand. The percentage of DMSO changed with every addition up to a maximum of about 5% in the sample solution, so the DMSO was neglected in the blank subtraction. To achieve a good assembly of the dyes to the template strands 15 equivalents were added in total, in which 10 equivalents were theoretically needed to gain a 100% assembly to T_10_ and (dA)_10_, respectively. The oligonucleotide T_10_ consists of 10-mer oligothymidilate, (dA)_10_ consists of 10-mer oligo-2‘-deoxyadenylate. The final concentrations were 3 µM template and 45 µM dye. To enable an assessment about the assembly, also a negative sample was prepared in every case with 45 µM dye and without template strand and both samples were centrifuged at 14000 rpm for 20 min. This excludes the solubility of the dyes in water with 5% DMSO. As shown by previous achievements of our working group, an assessment can be enabled about the assembly by a look at the absorption of the centrifuged samples.

#### Confocal imaging

The protocol, established previously [26], was adapted for imaging of compounds **4** or **5**. Briefly, human HeLa 21.4 cells were cultured for 48 h after seeding in Petri dishes with glass bottom (MaTek), reaching approximately 50% confluency. The cells were grown in Dulbeco’s minimal essential medium (DMEM) with 5% FCS, at 5% CO_2_ and 37 °C. The same medium was used to perform all microscopy experiments. Directly before imaging the cells were incubated with 500 nM of **4** or **5** added to the imaging medium. Stock solutions of these compounds were freshly prepared in DMSO (200 µM). Additionally, mitochondria of live cells were labelled (where indicated) with MitoTracker^®^ Red (Thermofisher, Poland) by incubation with 50 nM of the dye for 15 minutes. The imaging was performed using a LSM 780 confocal system (Zeiss), equipped with an AxioObserver Z1 inverted microscope, a 63× oil immersion objective (NA 1.4), a 355 nm DPSS laser (50 mW), a 561 nm DPSS laser (20 mW), and a multi-anode PMT (32 elements). The luminescence spectrum was registered from single confocal sections (pinhole set to 1 Airy unit), in the 395–685 nm range, with 4.2 nm spectral precision, using 355 nm (5.0% of nominal power) excitation. Where indicated, detector elements were combined into detection bands corresponding to 395–475 nm (**4** and **5,** excitation 355 nm) and 568–685 nm (MitoTracker^®^ Red, excitation 561 nm). The luminescence and transmitted light images were collected with 0.4 µs pixel dwell time (2 × line averaging) and a pixel size of 0.055 µm.

### Synthesis

Compound **1** has been obtained according to literature [25].

Compound **1**. ^1^H NMR (600 MHz, DMSO-*d*_6_) δ 8.82 (d, *J*_H,H_ = 9.6 Hz, 1H, Pyr), 8.53 (d, *J*_H,H_ = 7.8 Hz, 1H, Pyr), 8.36 (q, *J*_H,H_ = 7.8 Hz, 3H, Pyr), 8.31 (t, *J*_H,H_ = 10.2 Hz, 2H, Pyr), 8.22 (d, *J*_H,H_ = 8.4 Hz, 1H, Pyr), 8.13 (t, *J*_H,H_ = 7.8 Hz, 1H, Pyr), 4.06 (t, *J*_H,H_ = 6.0Hz, 2H, CH_2_), 3.79 (t, *J*_H,H_ = 6.0Hz, 2H, CH_2_); ^13^C NMR (150 MHz, CDCl_3_) δ 201.2, 133.5, 131.6, 130.7, 130.0, 129.7, 129.6, 128.5, 127.2, 126.98, 126.91, 126.7, 126.2, 124.5, 124.4, 124.1, 123.5, 44.4, 40.2; MS (EI, 70 eV) *m*/*z*: 294 ([M + 2]^+^), 292 (M^+^), 256 (acryloylpyrene^+^); FTIR (KBr) ν: 3122, 3109, 3053, 3036, 1664 (C=O), 847 cm^−1^.

#### General procedure for the synthesis of **2** and **3**

A mixture of 3-chloropropionylpyrene (293 mg, 1.0 mmol) and Et_3_N (278 μL) in DMF (20 mL) was vigorously stirred at room temperature for 20 min. Then, the appropriate nucleobase (1 equiv) was added (in a solid state) and the mixture was stirred at a temperature of 75 °C for 5 h. Subsequently, the solvent was evaporated to dryness and the residue subjected to column chromatography on SiO_2_ (**2,** eluent chloroform/methanol 50:0.5 (v/v), **3**, eluent chloroform/methanol 50:1 (v/v)). Crystallization from chloroform/*n*-hexane afforded pure **2** (54%, 208 mg, yellow solid), or **3** (56%, 220 mg, yellow solid).

Compound **2.** ^1^H NMR (600 MHz, DMSO-*d*_6_) δ 11.20 (s, 1H, NH), 8.86 (d, *J*_H,H_ = 9.3 Hz, 1H, Pyr), 8.59 (d, *J*_H,H_ = 8.1 Hz, 1H, Pyr), 8.42–8.32 (m, 5H, Pyr), 8.26 (d, *J*_H,H_ = 8.9 Hz, 1H, Pyr), 8.16 (t, *J*_H,H_ = 7.6 Hz, 1H, Pyr), 7.60 (d, *J*_H,H_ = 1.2 Hz, 1H, H-6 thymine), 4.13 (t, *J*_H,H_ = 6.9 Hz, 2H, CH_2_), 3.70 (t, *J*_H,H_ = 6.9 Hz, 2H, CH_2_), 1.72 (d, *J*_H,H_ = 1.2 Hz, 1H, CH_3_ thymine); ^13^C NMR (150 MHz, CDCl_3_) δ 202.3, 164.3, 151.0, 141.9, 131.4, 130.7, 130.0, 129.7, 129.6, 128.5, 127.2, 126.8, 126.7, 126.2, 124.5, 124.4, 124.1, 123.5, 108.3, 44.0, 40.7, 12.0; MS (EI, 70 eV) *m*/*z*: 382 (M^+^), 256 (acryloylpyrene^+^), 126 (thymine^+^); FTIR (KBr) ν: 3177, 3049, 2925, 2822, 1689 (C=O), 1652 (C=O), 1626 (C=O) cm^−1^; anal. calcd for C_24_H_18_N_2_O_3_ + CH_2_Cl_2_: C, 64.25; H, 4.31; N, 5.99; found: C, 64.03; H, 4.33; N, 5.93.

Compound **3**. ^1^H NMR (600 MHz, DMSO-*d*_6_) δ 8.80 (d, *J*_H,H_ = 9.3 Hz, 1H, Pyr), 8.56 (d, *J*_H,H_ = 8.1 Hz, 1H, Pyr), 8.40–8.31 (m, 5H, Pyr), 8.24 (d, *J*_H,H_ = 9.0 Hz, 1H, Pyr), 8.23 (s, 1H, H-2 adenine), 8.15 (d, *J*_H,H_ = 7.62 Hz, 1H, Pyr), 8.14 (s, 1H, H-8 adenine), 7.15 (s, 2H, NH_2_ adenine), 4.66 (t, *J*_H,H_ = 6.72 Hz, 2H, CH_2_), 3.98 (t, *J*_H,H_ = 6.72 Hz, 2H, CH_2_); ^13^C NMR (150 MHz, DMSO-*d*_6_) δ 201.8, 156.1, 152.5, 149.7, 141.2, 133.5, 131.2, 130.7, 129.9, 129.7, 129.6, 128.6, 127.24, 127.20, 126.8, 126.7, 124.5, 124.4, 123.5, 118.9, 41.3, 39.1; ESIMS *m*/*z*: 392 (M + H^+^); FTIR (KBr) ν: 3300 (N-H), 3142 (N-H), 3041, 2923, 1670 (C=O), 1603 (N-H bending), 1214, 846 cm^−1^; anal. calcd for C_24_H_17_N_5_O: C, 73.64; H, 4.38; N, 17.89; found: C, 73.57; H, 4.40; N, 17.77.

#### Synthesis of compound **4**

To a stirred solution of compound **2** (268 mg, 0.7 mmol) in THF (20 mL) was added NaBH_4_ (38 mg, 1.0 mmol) at room temperature. After 30 minutes the reaction mixture was poured into water (≈30 mL), extracted with chloroform (≈40 mL), dried over MgSO_4_, filtered and evaporated to dryness. The residue was subjected to column chromatography on SiO_2_ (eluent chloroform/methanol 50:2 (v/v)). Crystallization from chloroform/*n*-hexane gave the pure compound **4** as colourless solid in 91% yield (245 mg).

^1^H NMR (600 MHz, DMSO-*d*_6_) δ 11.14 (s, 1H, NH), 8.38 (d, *J*_H,H_ = 9.3 Hz, 1H, Pyr), 8.30–8.27 (m, 3H, Pyr), 8.25 (d, *J*_H,H_ = 7.9 Hz, 1H, Pyr), 8.20 (d, *J*_H,H_ = 9.3 Hz, 1H, Pyr), 8.15 (d, *J*_H,H_ = 0.8 Hz, 2H, Pyr), 8.06 (t, *J*_H,H_ = 7.6 Hz, 1H, Pyr), 7.49 (d, *J*_H,H_ = 1.0 Hz, 1H, H-6 thymine), 5.73 (d, *J*_H,H_ = 4.2 Hz, 1H, OH), 5.69–5.66 (m, 1H, C*H*(OH)), 3.96–3.92 (m, 1H, CH_2_), 3.90–3.86 (m, 1H, CH_2_), 2.22–2.17 (m, 1H, CH_2_), 2.15–2.09 (m, 1H, CH_2_), 1.69 (d, *J*_H,H_ = 1.0 Hz, 3H, CH_3_ thymine); ^13^C NMR (150 MHz, DMSO-*d*_6_) δ 164.3, 151.0, 141.7, 139.4, 131.0, 130.3, 129.9, 127.5, 127.3, 126.9, 126.7, 126.2, 125.2, 125.0, 124.2, 124.1, 123.8, 122.8, 108.4, 67.2, 45.5, 38.0, 26.4, 11.9; MS (EI, 70 eV) *m*/*z*: 384 (M^+^), 366 (M^+^ − H_2_O); FTIR (KBr) ν: 3421, 3041, 2925, 1671 (C=O broad) cm^−1^; anal. calcd for C_24_H_20_N_2_O_3_: C, 74.98; H, 5.24; N, 7.29; found: C, 75.01; H, 5.52; N, 7.02.

#### Synthesis of compound **5**

To a stirred solution of compound **3** (196 mg, 0.5 mmol) in THF (20 mL) was added LiAlH_4_ (0.5 mmol, 0.5 mL) at room temperature. After 10 minutes of stirring the reaction mixture was poured into water (≈30 mL), extracted with chloroform (≈40 mL), dried over MgSO_4_, filtered and evaporated to dryness. The residue was subjected to column chromatography on SiO_2_ (eluent chloroform/methanol 50:2 (v/v)). Crystallization from chloroform/*n*-pentane afforded the pure compound **5** as colourless solid in 85% yield (167 mg).

^1^H NMR (600 MHz, DMSO-*d*_6_) δ 8.28–8.26 (m, 4H, Pyr, adenine), 8.18 (s, 1H, H-2 adenine), 8.17 (d, *J*_H,H_ = 9.6 Hz, 1H, Pyr), 8.15–8.14 (m, 2H, Pyr, adenine), 8.10 (d, *J*_H,H_ = 9.6 Hz, 1H, Pyr), 8.05 (t, *J*_H,H_ = 7.80 Hz, 1H, Pyr), 7.16 (s, 2H, NH_2_ adenine), 8.84 (d, *J*_H,H_ = 4.2 Hz, 1H, OH), 5.64–5.61 (m, 1H, C*H*(OH)), 4.50–4.45 (m, 1H, CH_2_), 4.43–4.39 (m, 1H, CH_2_), 2.52–2.46 (m, 1H, CH_2_), 2.34–2.28 (m, 1H, CH_2_); ^13^C NMR (150 MHz, DMSO-*d*_6_) 156.1, 152.4, 149.7, 141.19, 141.18, 139.4, 130.9, 130.2, 129.9, 127.5, 127.3, 126.9, 126.6, 126.2, 125.2, 125.0, 124.2, 124.0, 123.8, 122.6, 119.0, 66.9, 40.9, 38.9; ESIMS *m*/*z*: 393 (M + H^+^); FTIR (KBr) ν: 3275 (N-H), 3132 (N-H), 3041, 2949, 2923, 2854, 1701 (N-H), 1613 (N-H), 1302, 852, 843 cm^−1^; anal. calcd for C_24_H_19_N_5_O: C, 73.27; H, 4.87; N, 17.80; found: C, 73.05; H, 5.09; N, 17.59.

### Single-crystal X-ray structure analysis

A good quality single-crystal of **2** was selected for the X-ray diffraction experiments at *T* = 100(2) K. Diffraction data were collected on an Agilent Technologies SuperNova Dual Source diffractometer with Cu Kα radiation (λ = 1.54184 Å) using the CrysAlis RED software [41]. The analytical numerical absorption correction using a multifaceted crystal model based on expressions derived by R.C. Clark & J.S. Reid [42] implemented in the SCALE3 ABSPACK scaling algorithm, was applied [41]. The structural determination procedure was carried out using the SHELX package [43]. The structures were solved with direct methods and then successive least-square refinement was carried out based on the full-matrix least-squares method on *F*^2^ using the XLMP program [43]. The H-atom linked to the N-atom was located on the Fourier difference map and refined as riding with *U*_iso_(H) = 1.2*U*_eq_(N). Other H-atoms were positioned geometrically, with the C–H bond length equal to 0.93, 0.96, 0.97 and 0.98 Å for the aromatic, methyl and methylene and methine H atoms, respectively, and constrained to ride on their parent atoms with *U*_iso_(H) = x*U*_eq_(C), where x = 1.2 for the aromatic, methylene and methine H atoms, and x = 1.5 for the methyl H atoms. All presented molecular interactions were found using the PLATON program [44]. The figures for this publication were prepared using ORTEP-3 and Mercury programs [45–46].

The CCDC 1555530 contains the supplementary crystallographic data for this paper. The data can be obtained free of charge from The Cambridge Crystallographic Data Centre via http://www.ccdc.cam.ac.uk/structures.

## Supporting Information

File 1 ^1^H NMR spectra, refinement data, and spectra in HeLa cells.
