# Supplementary material for: Pyrene–nucleobase conjugates: synthesis, oligonucleotide binding and confocal bioimaging studies
Source: Beilstein J Org Chem. 2017 Nov 28;13:2521–34. doi: 10.3762/bjoc.13.249 (PMC5727867; doi:10.3762/bjoc.13.249)
Supplement: File 1 — 1H NMR spectra, refinement data, and spectra in HeLa cells. [file Beilstein_J_Org_Chem-13-2521-s001.pdf]

Supporting Information

for

Pyrene–nucleobase conjugates: synthesis, oligonucleotide binding and confocal bioimaging studies

Artur Jabłoński[1], Yannic Fritz[2], Hans-Achim Wagenknecht[2], Rafał Czerwieniec[3], Tytus Bernaś[4], Damian Trzybiński[5], Krzysztof Woźniak[5] and Konrad Kowalski[*1]

Address: [1]Faculty of Chemistry, Department of Organic Chemistry, University of Łódź, Tamka 12, PL-91403 Łódź, Poland, [2]Institute of Organic Chemistry, Karlsruhe Institute of Technology, Fritz-Haber-Weg 6, 76131 Karlsruhe, Germany, [3]Universität Regensburg, Institut für Physikalische und Theoretische Chemie, Universitätsstraße 31, D-93040 Regensburg, Germany, [4]Nencki Institute of Experimental Biology, Polish Academy of Sciences, ul. Pasteura 3, 02-093 Warsaw, Poland and [5]Faculty of Chemistry, Biological and Chemical Research Centre, University of Warsaw, Żwirki and Wigury 101, 02-089 Warszawa, Poland

[*]Corresponding author
Email: Konrad Kowalski - kondor15@wp.pl

^{1}H NMR spectra, refinement data, spectra in HeLa cells

Contents

**Figure S1** ^{1}H NMR of compound **1**	**S3**
**Figure S2** ^{1}H NMR of compound **2**	**S4**
**Figure S3** ^{1}H NMR of compound **3**	**S5**
**Figure S4** ^{1}H NMR of compound **4**	**S6**
**Figure S5** ^{1}H NMR of compound **5**	**S7**
**Table S1** Crystal and structure refinement data for compound **2**	**S8**
**Table S2** Bond lengths, valence and torsion angles in **2**	**S9**
**Table S3** The geometry of hydrogen bonds in **2**	**S12**
**Table S4** The geometry of halogen bond in **2**	**S13**
**Figure S6** The arrangement of molecules in the crystal of **2**	**S14,S15**
**Figure S7** Fluorescence emission spectra of **4** and **5** in HeLa cells	**S16**

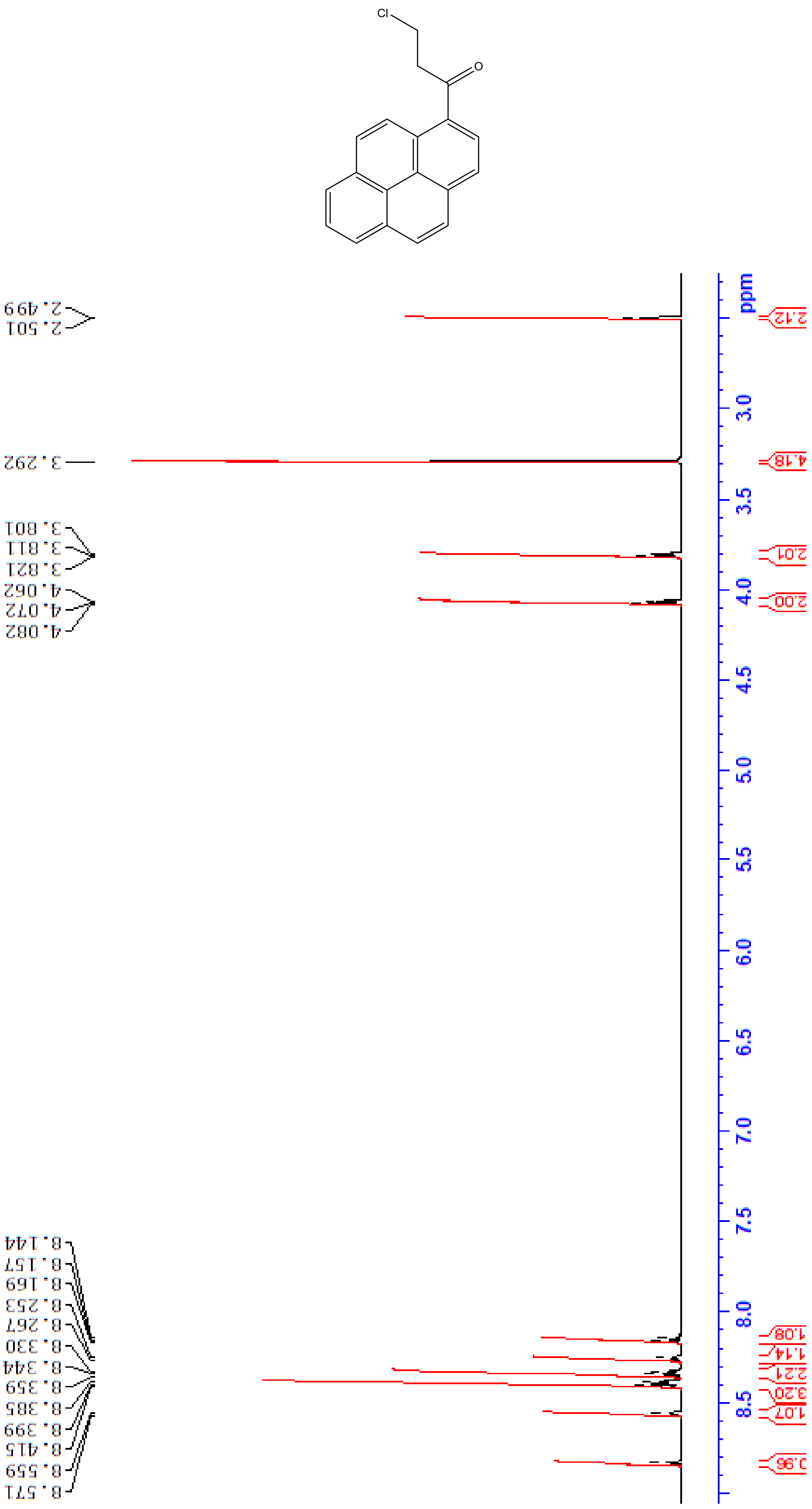

Figure S1: ^{1}H NMR of compound **1**.

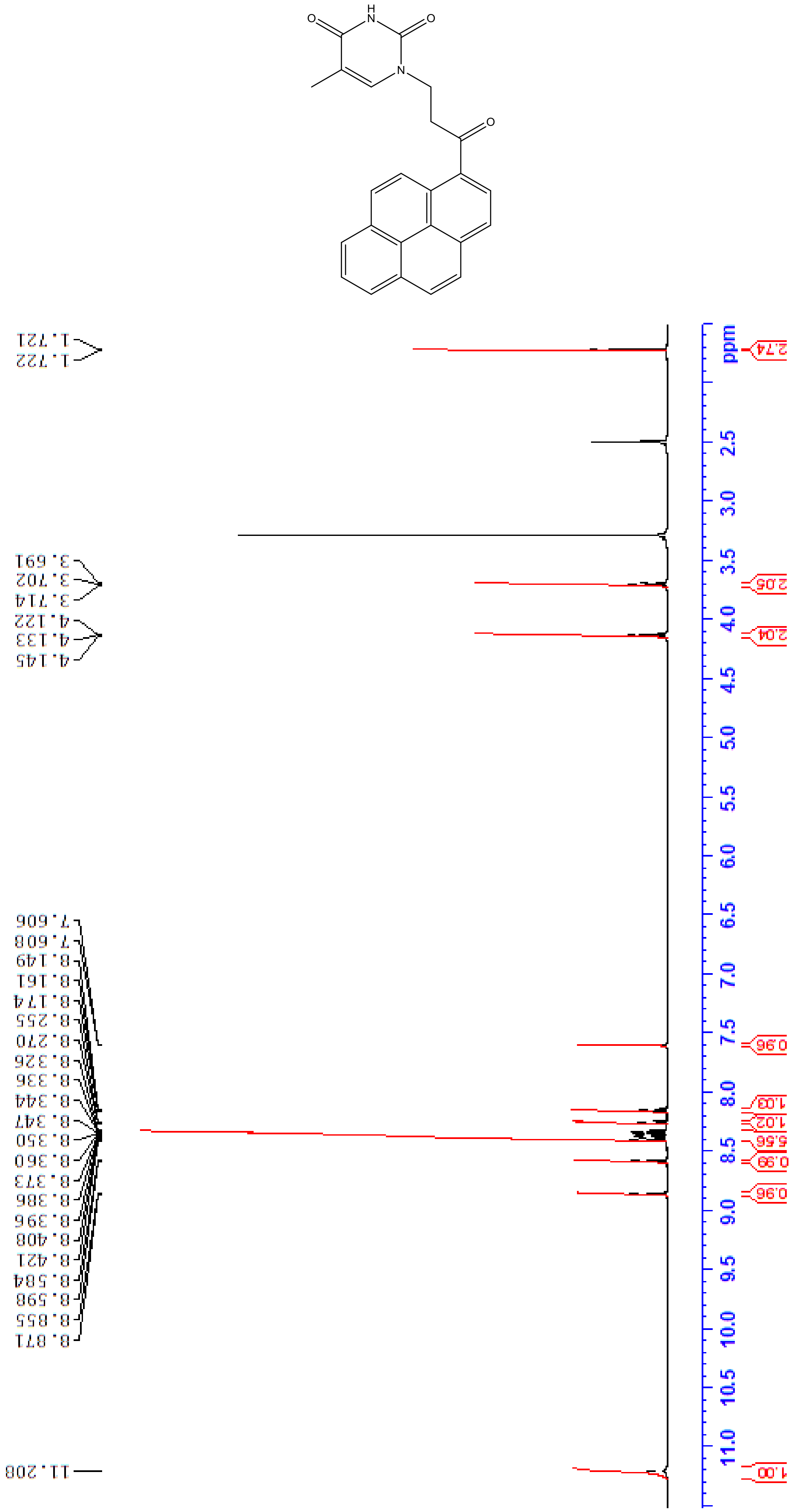

Figure S2: ^{1}H NMR of compound **2**.

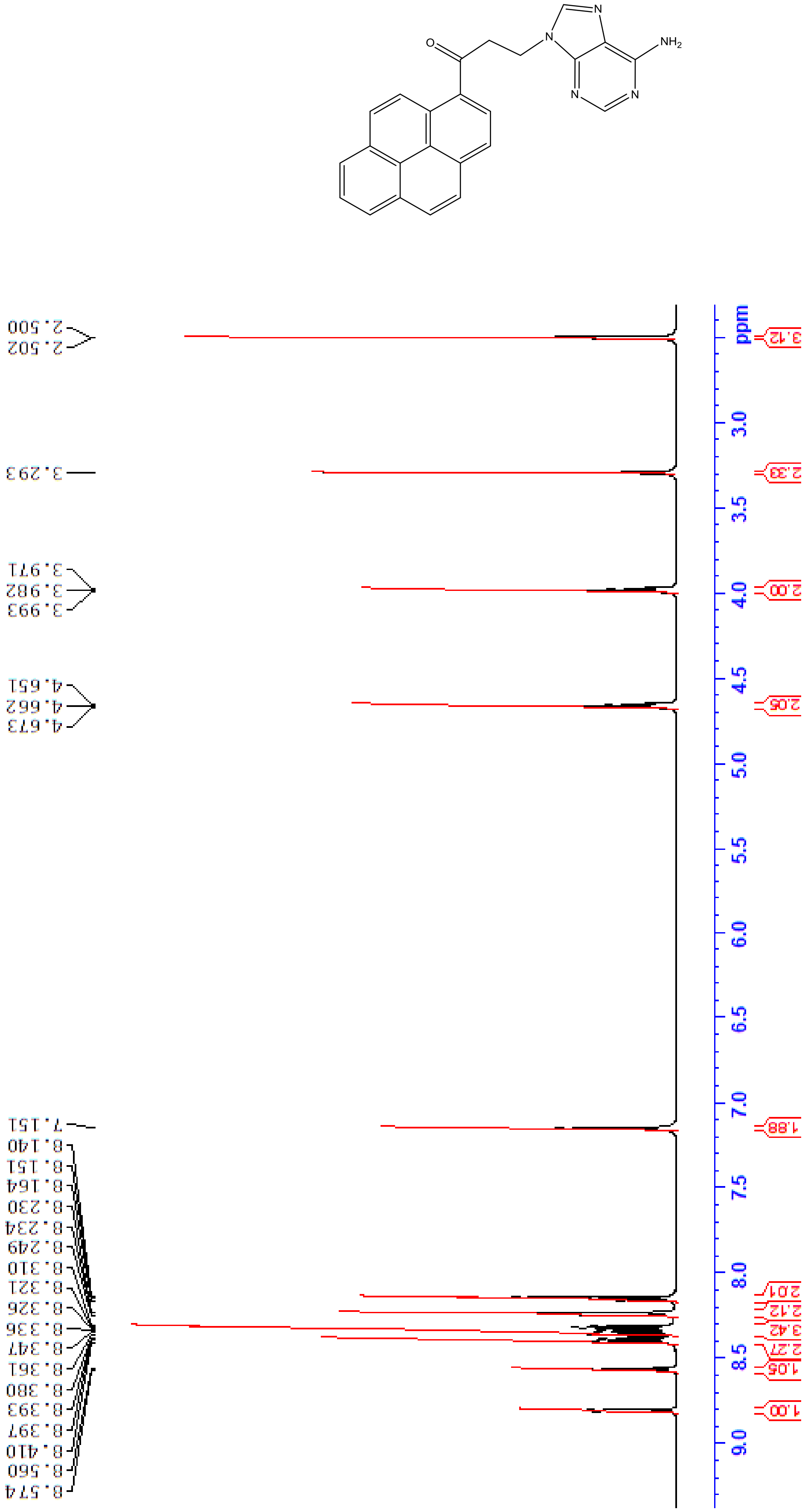

Figure S3: ^{1}H NMR of compound **3**.

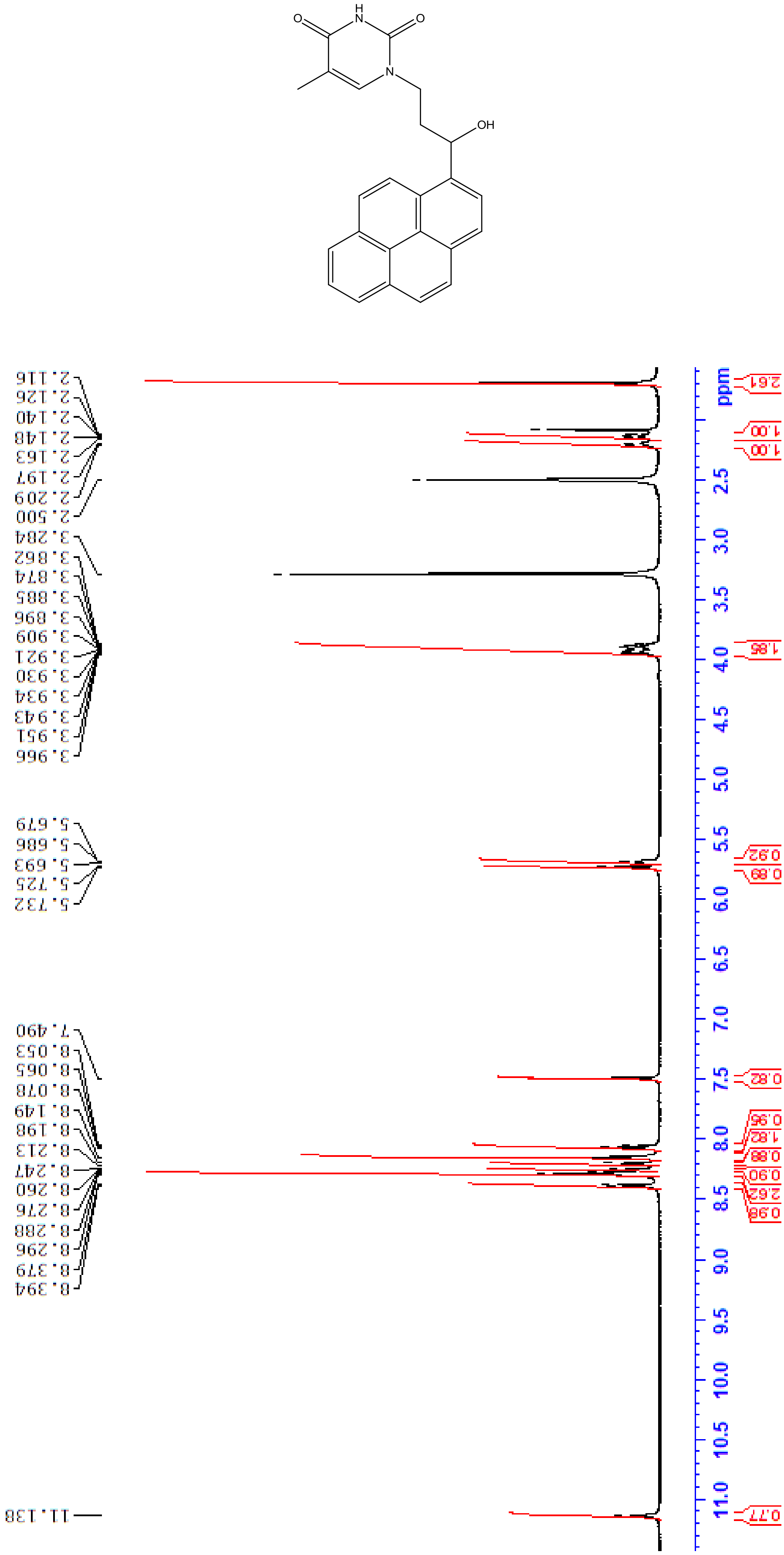

Figure S4: ^{1}H NMR of compound **4**.

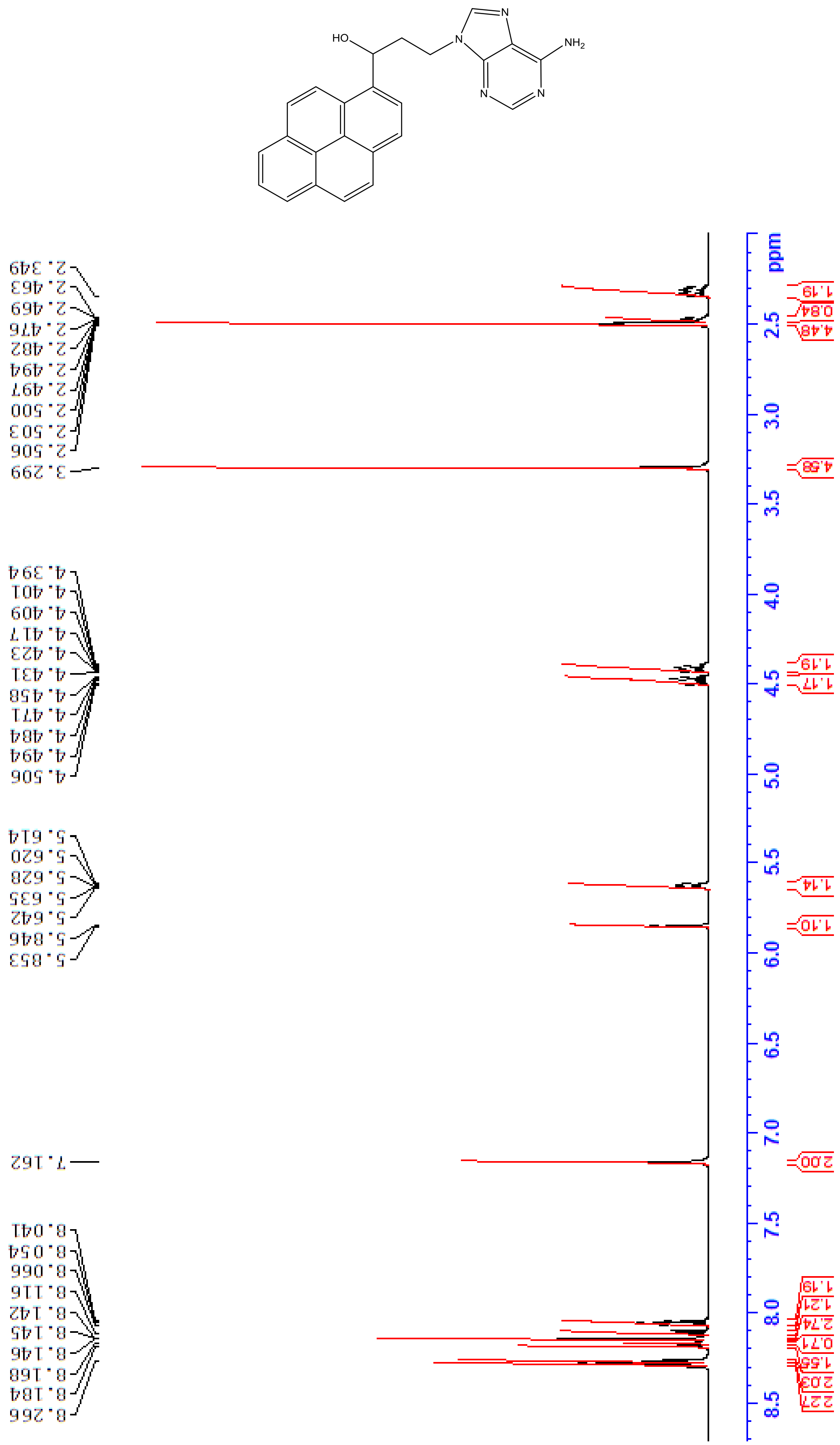

Figure S5: ^{1}H NMR of compound **5**.

Table S1: Crystallographic data and structural refinement details of **2**.

**Compound**	**2**
Empirical formula	$C_{24}H_{18}N_2O_3$, $CHCl_3$
Formula weight	501.77
Temperature/K	100(2)
Crystal system	Monoclinic
Space group	*I*2/*a*
a/Å	21.1411(2)
b/Å	5.2389(5)
c/Å	39.4030(3)
β/°	90.0816(9)
Volume/Å^3	4362.45(7)
Z	8
ρ_{calc}/g/cm^3	1.528
μ/mm^{-1}	4.078
F(000)	2064
Crystal size/mm^3	0.42 × 0.24 × 0.05
Radiation	Cu*K*α (λ = 1.54184 Å)
2Θ range for data collection/°	2.24 to 74.49
Index ranges	$-26 \leq h \leq 24$, $-6 \leq k \leq 6$, $-49 \leq l \leq 49$
Reflections collected	77735
Independent reflections	4482 [R_{int} = 0.069]
Data/restraints/parameters	4482/0/302
Goodness-of-fit on F^2	1.09
Final *R* indexes [I>=2σ (I)]	R_1 = 0.0426, wR_2 = 0.1220
Final *R* indexes [all data]	R_1 = 0.0439, wR_2 = 0.1236
Largest diff. peak/hole / e Å^{-3}	0.59/-0.64

Table S2: Bond lengths, valence and torsion angles in in the chloroform solvate of **2** (Å, °).

C1—C2	1.428(2)
C15—C16	1.433(2)
C1—C14	1.403(2)
C17—C18	1.520(2)
C1—C17	1.501(2)
C17—O26	1.223(2)
C2—C3	1.438(2)
C2—C15	1.427(2)
C3—C4	1.360(2)
C4—C5	1.428(3)
C19—N20	1.476(2)
C5—C6	1.405(2)
C21—N20	1.370(2)
C5—C16	1.415(2)
C21—N22	1.372(2)
C21—O27	1.233(2)
C6—C7	1.385(3)
C23—C24	1.446(3)
C23—N22	1.389(2)
C7—C8	1.384(3)
C23—O28	1.225(2)
C24—C25	1.352(3)
C8—C9	1.399(2)
C24—C29	1.495(3)
C9—C10	1.433(3)
C9—C16	1.422(2)
C25—N20	1.380(2)
C10—C11	1.350(3)
C11—C12	1.437(2)
N22—H22	0.82(2)
C12—C13	1.399(2)
C12—C15	1.422(2)
C30—Cl31	1.754(2)
C30—Cl32	1.756(2)
C13—C14	1.381(2)
C30—Cl33	1.758(2)
C2—C1—C17	123.28(15)
C9—C16—C15	120.34(16)
C14—C1—C2	118.79(15)
C1—C17—C18	116.90(14)
C14—C1—C17	117.93(15)
O26—C17—C1	123.78(15)
C1—C2—C3	124.04(16)
O26—C17—C18	119.31(15)
C15—C2—C1	118.57(15)
C15—C2—C3	117.34(15)
C4—C3—C2	121.92(16)
C19—C18—C17	112.97(14)
C3—C4—C5	121.60(17)
C6—C5—C4	122.18(17)
C6—C5—C16	119.38(16)
N20—C19—C18	110.72(13)
C16—C5—C4	118.43(16)
C7—C6—C5	120.57(17)
N20—C21—N22	115.61(14)
O27—C21—N20	122.60(15)
O27—C21—N22	121.79(15)
N22—C23—C24	114.79(15)
O28—C23—C24	125.09(16)

O28—C23—N22	120.12(16)
C7—C8—C9	120.78(17)
C23—C24—C29	117.35(17)
C25—C24—C23	118.30(16)
C8—C9—C10	121.56(17)
C25—C24—C29	124.34(18)
C8—C9—C16	119.27(17)
C16—C9—C10	119.16(16)
C24—C25—N20	123.48(17)
C11—C10—C9	120.89(17)
C10—C11—C12	121.28(17)
C13—C12—C11	121.42(16)
C13—C12—C15	118.87(16)
C21—N20—C19	119.48(14)
C15—C12—C11	119.69(16)
C21—N20—C25	120.79(14)
C25—N20—C19	119.64(14)
C14—C13—C12	120.45(16)
C21—N22—C23	126.95(15)
C13—C14—C1	122.33(16)
Cl31—C30—Cl32	111.30(12)
C2—C15—C16	120.50(15)
Cl31—C30—Cl33	110.81(11)
C12—C15—C2	120.90(15)
C12—C15—C16	118.59(15)
Cl32—C30—Cl33	110.13(11)
C5—C16—C9	119.46(16)
C5—C16—C15	120.19(15)
C1—C2—C3—C4	−176.24(18)
C12—C15—C16—C5	178.84(16)
C1—C2—C15—C12	−2.4(2)
C12—C15—C16—C9	−0.2(2)
C1—C2—C15—C16	176.54(15)
C13—C12—C15—C2	2.3(2)
C1—C17—C18—C19	−179.70(14)
C13—C12—C15—C16	−176.66(15)
C2—C1—C14—C13	2.6(3)
C14—C1—C2—C3	177.26(17)
C2—C1—C17—C18	165.88(15)
C14—C1—C2—C15	0.0(2)
C2—C1—C17—O26	−15.5(3)
C14—C1—C17—C18	−13.6(2)
C2—C3—C4—C5	−0.2(3)
C14—C1—C17—O26	165.03(16)
C2—C15—C16—C5	−0.1(2)
C15—C2—C3—C4	1.1(3)
C2—C15—C16—C9	−179.19(15)
C15—C12—C13—C14	0.3(3)
C3—C2—C15—C12	−179.85(16)
C16—C5—C6—C7	−1.3(3)
C3—C2—C15—C16	−0.9(2)
C16—C9—C10—C11	0.8(3)
C3—C4—C5—C6	178.24(19)
C17—C1—C2—C3	−2.2(3)
C3—C4—C5—C16	−0.9(3)
C17—C1—C2—C15	−179.48(15)
C4—C5—C6—C7	179.59(18)
C17—C1—C14—C13	−177.90(15)
C4—C5—C16—C9	−179.89(16)
C17—C18—C19—N20	−178.06(13)

C4—C5—C16—C15	1.0(3)
C18—C19—N20—C21	−88.98(18)
C5—C6—C7—C8	0.4(3)
C18—C19—N20—C25	87.55(19)
C6—C5—C16—C9	1.0(3)
C23—C24—C25—N20	−0.2(3)
C6—C5—C16—C15	−178.14(16)
C24—C23—N22—C21	−1.0(3)
C6—C7—C8—C9	0.8(3)
C24—C25—N20—C19	−178.73(19)
C7—C8—C9—C10	179.43(18)
C24—C25—N20—C21	−2.2(3)
C7—C8—C9—C16	−1.1(3)
C29—C24—C25—N20	179.6(2)
C8—C9—C10—C11	−179.65(18)
N20—C21—N22—C23	−1.3(3)
C8—C9—C16—C5	0.2(3)
N22—C21—N20—C19	179.35(14)
C8—C9—C16—C15	179.30(16)
N22—C21—N20—C25	2.9(2)
C9—C10—C11—C12	0.9(3)
N22—C23—C24—C25	1.7(3)
C10—C9—C16—C5	179.72(16)
N22—C23—C24—C29	−178.1(2)
C10—C9—C16—C15	−1.2(3)
O26—C17—C18—C19	1.6(2)
C10—C11—C12—C13	176.22(17)
O27—C21—N20—C19	−0.8(3)
C10—C11—C12—C15	−2.4(3)
O27—C21—N20—C25	−177.29(17)
C11—C12—C13—C14	−178.36(16)
O27—C21—N22—C23	178.89(17)
C11—C12—C15—C2	−179.06(16)
O28—C23—C24—C25	−178.38(19)
C11—C12—C15—C16	2.0(2)
O28—C23—C24—C29	1.8(3)
C12—C13—C14—C1	−2.8(3)
O28—C23—N22—C21	179.08(16)

Table S3: The geometry of hydrogen bonds in **2**.

D–H	A	d(D···A) (Å)	< D–H···A (°)
N22–H22	O27^{i}	2.882(2)	174(3)
C3–H3	O26*	2.879(2)	126
C14–H14	O27^{ii}	3.324(2)	162
C18–H18A	O28^{iii}	3.309(2)	161
C18–H18B	O27^{ii}	3.251(2)	143
C25–H25	O26^{iv}	3.442(2)	170

Symmetry codes: (i) –x + 1/2, –y + 7/2, –z + 3/2; (ii) x, y – 1/2, z; (iii) –x + 1/2, –y + 5/2, –z + 3/2; (iv) –x + 1, y – 1/2, –z + 3/2; (*) intramolecular interaction.

Table S4: The geometry of halogen bond in **2**.

**D–X**	**A**	**d(X···A) (Å)**	**d(D···A) (Å)**	**< D–X···A (°)**
C30–Cl31	O28	2.972(2)	4.644(2)	157.9(2)

In the crystal, inversely oriented molecules of **2** are linked by N–H···O hydrogen bonds, through $R^2_2(8)$ synthon, into pair (N22–H22···O27, d(D···A) = 2.882(2) Å, < D–H···A = 174(3)°) (Fig. S6a). Each molecule of **2** in such formed dimer is further incorporated in the Cl···O halogen bond with neighboring molecule of chloroform (C30–Cl31···O28, d(X···A) = 2.972(2) Å, < D–X···A = 157.9(3)°)(Fig. 6a). Adjacent dimers are thus participating in the network of weak C–H···O hydrogen bonds which is leading to formation of infinite 2D supramolecular framework, running along (001) plane (Fig. 6b). Detailed analysis performed by PLATON program (A. L. Spek, *Acta Crystallogr. Sect. D* 2009, *65*, 148–155.) indicates presence of the π-π and C=O·· π contacts between neighboring molecules of **2** within aforementioned supramolecular framework. The adjacent 2D-frameworks are oriented anti-parallel to themselves (Fig. 6c) and there is no direct interaction between them.

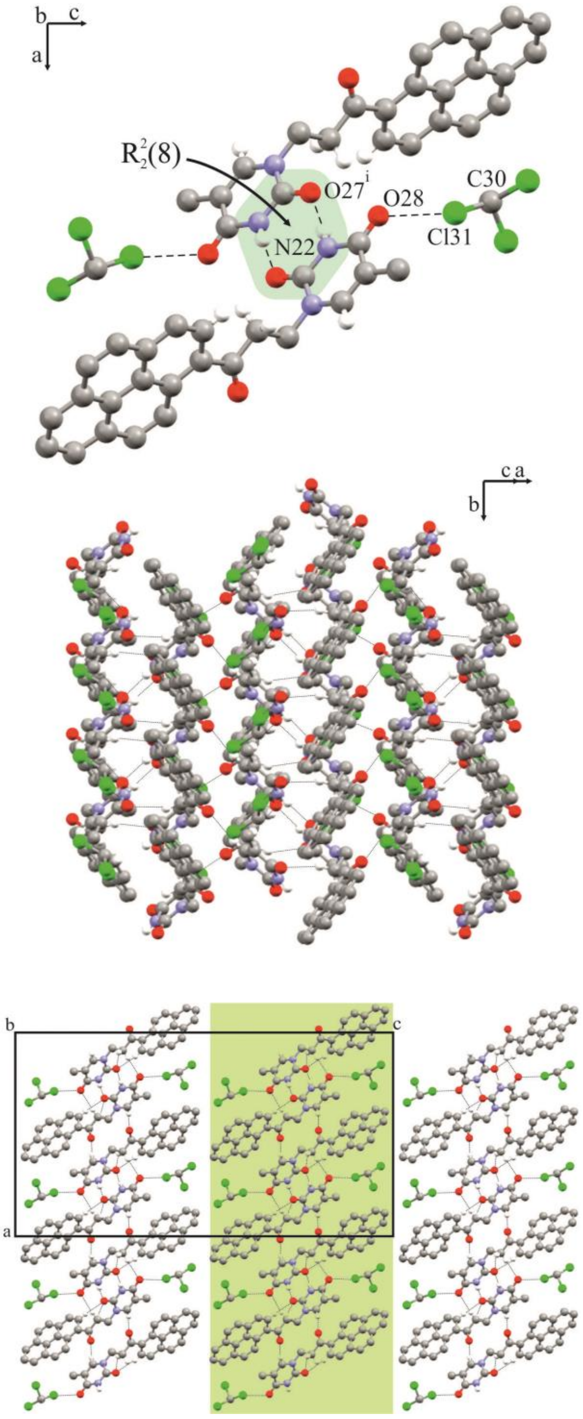

Figure S6: The arrangement of molecules in the crystal of the chloroform solvate of **2**, where: (a) dimer of molecules of **2** incorporated in the Cl···O halogen bonds with neighboring solvent molecules; (b) view from the side on 2D supramolecular framework formed by the adjacent molecules incorporated in halogen and hydrogen bonds; (c) general view on the structure (single 2D-framework is highlighted by green rectangle) along *b*-direction. The N–H···O, C–H···O and Cl···O intermolecular interactions are showed as a dashed lines. The H-atoms not participating in intermolecular interactions were omitted for clarity. Symmetry code: (i) –x + 1/2, –y + 7/2, –z + 3/2.

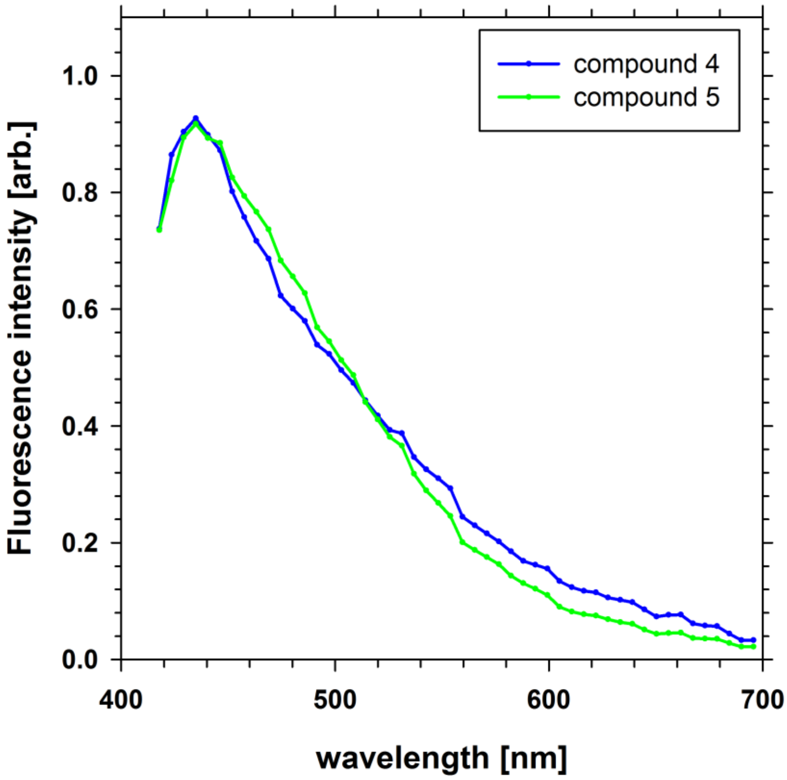

Figure S7: Fluorescence spectra **4** (blue line) and **5** (green line) measured in cytoplasm of live HeLa cells.
